# Traditional knowledge hiding in plain sight – twenty-first century ethnobotany of the Chácobo in Beni, Bolivia

**DOI:** 10.1186/s13002-017-0179-2

**Published:** 2017-10-10

**Authors:** Narel Y. Paniagua Zambrana, Rainer W. Bussmann, Robbie E. Hart, Araceli L. Moya Huanca, Gere Ortiz Soria, Milton Ortiz Vaca, David Ortiz Álvarez, Jorge Soria Morán, María Soria Morán, Saúl Chávez, Bertha Chávez Moreno, Gualberto Chávez Moreno, Oscar Roca, Erlin Siripi

**Affiliations:** 1 Herbario Nacionál de Bolivia, Universidad Mayor de San Andrés, Casilla 10077 Correo Central, La Paz, Bolivia; 2 Museo Nacional de Ciencias Naturales, Calle Ovidio Suarez 26, Cota Cota, La Paz, Bolivia; 3 William L. Brown Center, Missouri Botanical Garden, P.O. Box 299, St. Louis, MO 63166–0299 USA; 4 Instituto Linguistico Chácobo, Beni, Riberalta, Bolivia; 5 Comunidad Chácobo de Alto Ivón, Beni, Bolivia; 6 Comunidad Chácobo de Las Limas, Beni, Bolivia; 7 Comunidad Chácobo de Firmeza, Beni, Bolivia; 8 Comunidad Nueva Unión, Beni, Bolivia

**Keywords:** Traditional knowledge, Knowledge loss, Conservation, Indices

## Abstract

**Background:**

The Chácobo are a Panoan speaking tribe of about 1000 members (300+ adults) in Beni, Bolivia. Originally nomadic, the Chácabo were relocated to their current main location in the 1960s. Researchers have visited the Chácabo since 1911. A first more detailed anthropological report exists from the late 1960s, and ecological–ethnobotanical studies were conducted in the 1980s and 1990s. The presented work represents a complete ethnobotanical inventory of the entire adult Chácobo population, with interviews and plant collection conducted directly by Chácobo counterparts.

**Methods:**

Based on previous reports and our preliminary studies, we hypothesized that twenty-first century Chácobo plant use centered on income generation, and that traditional plant use related to household utensils, medicine and traditional crop varieties had almost disappeared. To test this hypothesis, we started the “Chácobo Ethnobotany Project,” training 10 indigenous Chácobo participants in ethnobotanical interview and plant collection techniques, in order to more fully document Chácobo knowledge and avoid the influence of foreign interviewers.

**Results:**

Our study found 331 useful plant species in 241genera of 95 plant families, with leaves, roots and bark being the most commonly used plant parts The comprehensive documentation that these methods enabled completely nullified our initial hypothesis of knowledge loss. Traditional crop varieties are still widely grown and traditional knowledge is alive. Moreover, it is being actively recuperated in certain domains by the younger generation. Most Chácobo know, and can name, traditional utensils and tools, although only the older generation has still the skills to manufacture them. While many Chácobo still know the names and uses of medicinal species, the younger generation is however often unsure how to identify them.

**Conclusions:**

In this paper we illustrate the complexity of perspectives on knowledge at different ages, and the persistence of knowledge over almost a century. We found that traditional knowledge was only partially affected by the processes of exposure to a market economy, and that different knowledge domains experienced different trends as a result of these changes. Overall knowledge was widely distributed, and we did not observe a directional knowledge loss.

We stress the importance to not directly conclude processes of knowledge loss, cultural erosion or acculturation when comparing the knowledge of different age groups.

## Background

The Chácobo tribe, living in Northeastern Bolivia, were first visited by the European traveler Erland Nordenskjöld in 1911 [1], followed by an anthropologist only in 1956, who published the last account of Chácobo life before the tribe came under the influence of American Evangelist missionaries [2]. The Summer Institute of Linguistics (SIL) worked with Chácobo communities from 1953 to 1980, and produced the first account of Chácobo linguistics [3], and an unpublished work on Chácobo customs, with a strong focus on evangelist development [4]. This account is in interesting juxtaposition to the writings of German anthropologist Kelm [5], who visited the Chácobo in 1970, in the middle of SIL rule. The SIL finally was replaced in 1980 by the Swiss Protestant mission. Missionary rule led to a profound change of lifestyle, and a permanent process of acculturation [6]. From 1983 to 84, Brian Boom (New York Botanical Garden) led the first ethnobotanical study of Chácobo, documenting their knowledge after almost 30 years of cultural change [7]. Boom did however base his work on the plants collected from a single 1 ha forest plot. In 1995 the Institut Franjáis d’Etudes Andines financed a re–survey of Boom’s plot, but the results were never released to the public, and a planned publication [8] existed in a single volume in the Institute’s main library in Lima. Muñoz et al. [9] published a study on anti–malarial plants used by the Chácobo. Given the availability of previous studies, the Chácobo are an outstanding possibility to study traditional knowledge over time.

Traditional knowledge (TK) has been recognized for its importance for the protection of ecosystem services and biodiversity [10, 11]. However, researchers and policymakers have equally expressed concern about its possible loss as societies modernize. A growing number of studies have reported changes and losses in TK (e.g. medical TK [12–15], nutritional TK [16], and agricultural TK [17–19]. The hypothesis that TK systems are able to adapt to external changes and internal pressures has discussed for some time (e.g., [20]). Traditional.

Knowledge is an important part of the adaptive capacity of many rural and indigenous communities that have been conserving biodiversity while enhancing livelihoods and adapting to disturbance and change [21, 22]. Few studies have however examined whether and how loss or alteration of TK in fact occurs [17, 23–25]. Consequently, our understanding of the resilience of TK systems and their ability to evolve and adapt is highly limited. The assumption of loss of TK, when younger people know less, is one of the common errors in ethnobotany [26]. This problem can be remedied when analyzing the effects of age and age cohorts separately [27, 28]. Not all TK domains might however be shared between generations, and as such there can be domains that would be more vulnerable to TK loss, and domains in which new knowledge is generated as an adaptation to environmental change [29].

Traditional knowledge is also seen as an important component in improving the management of natural resources [10, 20, 30] and practices relating to the protection of ecosystems and species [11]. Factors such as gender, age, ethnicity, birthplace, and level of education have been identified as important on an individual level [31–34]. Family size, integration into the market economy (e.g. sale of animals and agricultural products), or amount of material goods at family level (e.g., possessions of farm animals, tools, and transport) have been linked to the household levels [35, 36]. Access to commercial centers, and to health, education, electricity or water, as well as land tenure systems and settlement history have shown a greater relevance at the community level [37–39]. In the absence of a unifying theory or common research methods, it is however difficult to clearly recognize whether or not these patterns exist at broader scales [40]. Several studies have used literature metadata to analyze large–scale usage patterns of plants [41–43]. In many cases, however, comparisons are difficult to make, given the diversity of the objectives and methods employed.

Based on previous reports mentioned, and our own preliminary studies [44], we hypothesized that twenty-first century Chácobo plant use centered on income generation through collection of forest products and agricultural production, and that traditional plant use related to household artifacts and medicine, as well as traditional crop varieties, had almost disappeared. We also hypothesized that the “missionary generation” – the first age group growing up under restrictive evangelist rule, would report less TK than other age groups. Because access to markets and services has been reported as a major cause for TK loss [28], we also hypothesized that in villages most distant from the main market center (Riberalta), knowledge about the use of plants, and the number of useful species would be more homogeneously distributed through the generations [25], and expected that this TK distribution show different patterns when analyzing the different domains of knowledge about the use of plants [29].

To test our hypotheses, we started the “Chácobo Ethnobotany Project,” training 10 indigenous Chácobo participants in ethnobotanical interview and plant collection techniques, to comprehensively document contemporary Chácobo TK and avoid the limiting influence of foreign interviewers.

## Methods

### The study area –– The Chácobo and Pacahuara

The Chácobo belong to the Panoan linguistic group, which includes about twelve tribes (Chácobo, Pacahuara, Matis, Matses, Yaminahua, Ese Eja and others). At the end of the 1890s, the Chácobo lived as semi–nomadic hunters and cassava and maize cultivators, probably in two groups, one with six families and one with four, in north Bolivia, between Lake Roguagnado and the river Mamore, south of their current territory. During the rubber boom in the early 1900s, they were forced by more aggressive tribes to move north, where rubber tappers, who also brought disease and epidemics to the tribe, threatened them. While other tribes were enslaved to work in rubber stations, the Chácobo managed to avoid most of the outside influences. Their first permanent contact with the outside world occurred only in 1953 with members of the the Tribes Missions, and in 1954 the Bolivian government established an agency about 15 km from the current location of Puerto Limones. The missionary linguist Gilbert Prost arrived in 1955 under the auspices of the Summer Institute of Linguistics (SIL). According to [4] there were four Chácobo groups living between the Benicito and Yata rivers at that time, numbering about 200 people [7]. Prost and his wife continued to live among the Chácobo until 1980. In addition to translating the New Testament into Chácobo, they made some observations on cultural and linguistic practices [3, 4]. In 1964, Prost managed to buy a territory in the north of the Chácobo’s ancestral lands, forming the community of Alto Ivón, and most of the remaining population moved there. In 1965, the Bolivian government finally assigned 43,000 ha of land to the Chácobo, although this area was less than 10% of their original territory. The influence of the SIL caused profound cultural change among the Chácobo, including the reported abandonment of traditional costume and dances in 1969 [4].

**Fig. 1:**
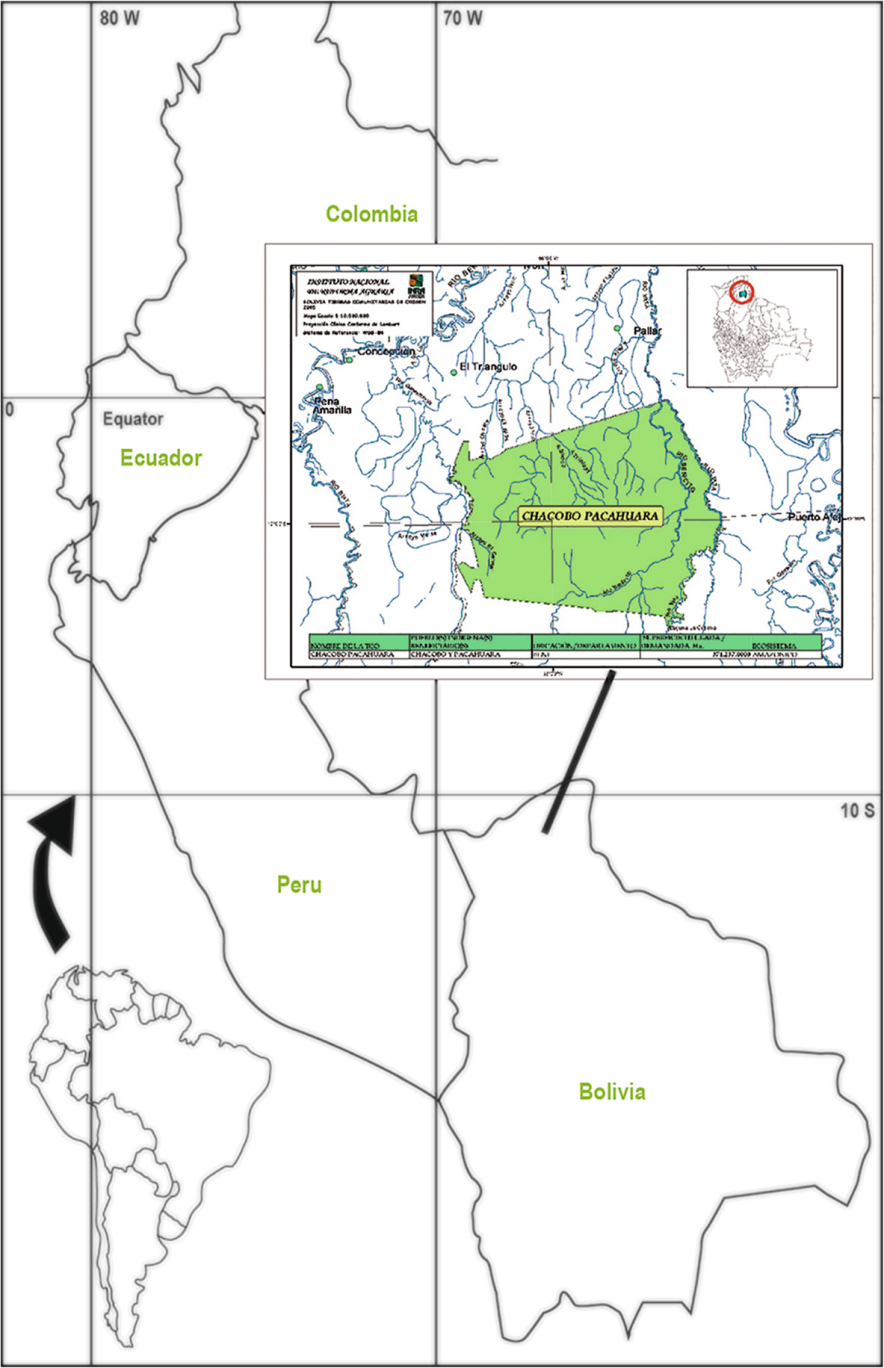
Chácobo territory 2013

The official indigenous organization of the Chácobo (Central Indígena de la Región Amazónica de Bolivia (CIRABO) estimates a current population of the Chácobo community of about 1000 people (350+ adults), with Alto Ivón as the largest settlement. The current territory of the tribe according to CIRABO encompasses 450,000 ha, and is roughly equivalent to the original extent of the tribe’s ancestral lands (Fig. 1). The elevation of the territory is about 200 m, and much of the vegetation can be classified as humid tropical Amazon rainforest. However, the territory encompasses also large tracts of periodically inundated savannas, dominated by *Mauritiella armata*, and large, drier, savanna areas with forest islands. The average annual temperature is 26.8 °C, with an average annual rainfall of 1560 mm. A distinct dry season lasts from June to November [7]. Today the Chácobo are governed by two indigenous organizations: The Capitanía Mayor Chácobo, closely linked to the evangelists, and the Chácobo– Pacahuara Association, recognized by the Central Indígena de la Región Amazónica de Bolivia (CIRABO), and supported by the Central de Pueblos Indigenas del Beni (CPIB) and the Confederacion de Pueblos Indigenas de Bolivia (CIDOB).

### Ethnobotanical and botanical collection

Our project explored the current traditional knowledge (TK) on plant use of the Chácobo and Pacahuara in Beni, Bolivia and had three goals: 1) to discover and document current traditional plant knowledge through interviews and surveys, 2) to inventory the current flora of the region, and 3) to repatriate the acquired knowledge as well as previous data to the community.

After obtaining consent from CIRABO, and before starting fieldwork, we conducted a community meeting in May 2013, involving representatives of all 27 villages in the Chácobo Territory, in order to obtain prior informed consent from all communities. This session included the repatriation of the results of previous studies [45–47]. In addition, during the project all available material on Chácobo plant use was translated to Spanish and repatriated [48]. The Chácobo community itself choose 12 local counterparts to be trained as ethnobotanical interviewers and plant collectors. In September 2013 we conducted a two–week workshop on ethnobiological methods and plant collection, training the 12 selected counterparts, 10 of which finally acted as interveiwers. Training was conducted directly in the field in the central village of Alto Ivón, and involved theoretical exercises (overview on methodology of interviews, collection and herbarium techniques), as well as extensive practical exercises (structuring and testing of questionnaires, test interviews among the participants, field interviews with local community members, plant collection in the field, preparation of herbarium specimens, plant and artifact collection in the local community, data–basing, and initial data analysis).

From November 2013 to May 2015, Chácobo interviewers collected ethnobotanical information from 301 Chácobo participants (150 women, 151 men, representing almost the entire adult Chácobo population), and over 1500 plant samples were collected. Prior to starting the interviews, every interviewer obtained prior oral informed consent from each participant. Chácobo participants were divided into five age classes (18–30 years old: 58 men, 52 women; 31–40 years old: 31 men, 36 women; 41–50 years old: 35 men, 36 women; 51–60 years old: 15 men, 7 women; and >60 years old: 12 men, 19 women). Because the study attempted to interview the whole adult Chácobo population, there was originally no emphasis on achieving a balanced age or gender distribution. All interviews were conducted at the homes of the participants by asking participants to freelist their plant knowledge following [49]. All plant uses were categorized following [49]. All interviews were preferably conducted in Chácobo. In a few cases where participants were not fully fluent in Chácobo, interviewers used Spanish as common language. The plant material was collected under permission from the Ministry of Environment and Water of the Plurinational State of Bolivia, and was identified and deposited at the National Herbarium of Bolivia (LPB) under the collection numbers of the Chácobo collectors. Nomenclature follows www.TROPICOS.org. Use descriptions were coded after the fact into subcategories and, for some analyses, into six major categories: fodder, fuel, medical, cultural, construction, tool, and food.

All work was carried out following the International Society for Ethnobiology Code of Ethics [50], and under the framework provided by the Nagoya Protocol on Access to Genetic Resources and Fair and equitable sharing of benefits arising from their use of the Convention on Biological Diversity, the Chácobo community retains the copyright of the traditional knowledge of all informants. Any commercial use of any of the information requires prior consensus with informants and communities, and an agreement on the distribution of benefits.

### Data analysis

The total number of unique species reported and unique uses reported for each use category were compared across communities, genders, and age groups (16–30, 31–40, 41–50, 51–60, and 61–82) for 292 informants (dropping for this analysis 8 informants for whom age was not indicated).

To gain a more nuanced look at how these qualities affected not only the number of reports but *which* species or uses were reported, we ordered informants using non–metric multi–dimensional scaling on distance matrices for plants and uses, and tested how well vectors (age) and factors (gender, ethnicity, community) fit the location of informants in the ordination, using the R package vegan [51]. We used similar methods with plant family fit onto an ordination from distance matrices of plant–use combinations to test whether plant family explains the uses to which plants are put.

We used Indicator Value [52], as implemented in the R package labdsv [53] to combine occurrence frequency and mean abundance of species and uses to elucidate species and uses that had higher fidelity to and/or relative abundance in certain age groups or genders. For this analysis, the *P* value is the probability of finding an equally high indicator value in random permutations. Species with significantly high indicator values had higher fidelity and relative abundance in certain age groups / genders (were ‘indicators’). We further compared age and gender groups by informant consensus factor (ICF) for each use category, calculated as the number of use reports minus the number of taxa over the number of use reports minus one: (Nur − Nt)/(Nur − 1). We also measured consensus on species uses by quantifying what proportion of each species’ mentions fall within a specific use category.

Plant species and plant family importance was ranked by four metrics: Community and Informant Cultural Importance (CIcom/CIinf) — the sum within species across all plant–uses of the number of informants (for CIinf) or communities (for CIcom) reporting a plant–use over the number of informants/communities reporting the plant; Diversity of Uses (Du) — the Shannon Index of uses [51]; and Use Value (UV), the number of reports of a species over total number of informants asked in a region [54].

To test whether greater knowledge of Chácobo language was associated with a more similar set of knowledge and / or a larger knowledge set of plants and uses, we used the ordination based on uses to examine whether interviewees who reported more Chácobo names tended to report a more similar set of uses, and used linear regression to test whether the number of Chácobo names reported was significantly greater for those who reported more species or more uses.

## Results

The availability of previous field data gives the unique opportunity to study the long–term change in knowledge of an indigenous group in the age of globalization. Our study found 331 useful plant species in 241genera of 95 plant families, with leaves, roots and bark being the most commonly used plant parts (Table 1).

**Table 1:** Plant species used by the Chácobo

FAMILY / SCIENTIFIC NAME	USES	VERNACULAR NAMES (Ch-Chácobo, Sp-Spanish)	Coll #
Amaranthaceae			
*Chenopodium ambrosioides* L.	MEDVET: Digestive system (Appendicitis, Leaf; Diarrhea, Leaf); General Ailments with Unspecific Symptoms (Chest pain, Leaf); Respiratory system (Bronchitis, Leaf)	Caré (Ch)	GOS 44, JSM 13, ORC 5
Amaryllidaceae			
*Allium cepa* L.	HUMFOOD: Food (Edible, Root); MEDVET: Infections and infestations (Tuberculosis, Root); Respiratory system (Cold and flu, Root)	Cebolla (Sp)	
*Allium sativum* L.	MEDVET: Cultural diseases and disorders (Bad air and scare - Ratëaina, Root); General Ailments with Unspecific Symptoms (Chest pain, Root); Infections and infestations (Malaria and fever, Root); Reproductive system and sex health (Menstrual pain, Root); Snakebites and Ray stings (Sankebites, Root)	Ajo (Sp)	
Anacardiaceae			
*Anacardium occidentale* L.	HUMFOOD: Food (Edible, Fruit); MEDVET: Dental health (Toothache, Seeds); Digestive system (Diarrhea, Bark, fruit, leaf and young leaf; Stomach ache, Leaf); Endocrine system (Diabetes, Bark; Liver pain, Leaf); General Ailments with Unspecific Symptoms (Vomit, Bark, fruit, leaf and young leaf); Skin and subcutaneous tissue (Puchichi, Leaf); Urinary system (Kidney infection, Bark)	Cayú (Sp)	BCM 1, GOS 14
*Astronium* sp.	FUEL: Firewood (Firewood - Caro, Trunk)	Mërabi (Ch)	MOV 38
*Mangifera indica* L.	HUMFOOD: Food (Edible, Fruit); MEDVET: Infections and infestations (Malaria and fever, Bark); UTEN&TOOL: Labour tools (Shovel, Trunk)	Manga (Sp)	
*Spondias venosa* C. Martius ex Colla	HUMFOOD: Food (Edible, Fruit)	Cedrillo (Sp)	CH1
*Spondias venulosa* C. Martius ex Colla	MEDVET: Musculo-skeletal system (Fractures, Bark)	Conserbilla de cuchi (Sp)	CH2
*Tapirira guianensis* Aubl.	CONST: Houses (Frame house, Trunk; House post- Jibamë, Trunk); MEDVET: Digestive system (Diarrhea, Bark, Stomach ache, Bark); Endocrine system (Gallbladder, Bark); General Ailments with Unspecific Symptoms (Vomit, Bark); Infections and infestations (Malaria and fever, Bark); Skin and subcutaneous tissue (Caracha, Bark; Skin fungus, Bark)	Chaxo nihi / Tarari / Xaba cano (Ch)	JSM 8, MSM 5, RBU 17827
Annonaceae			
*Annona hypoglauca* Mart.	HUMFOOD: Food (Edible, Fruit); MEDVET: Musculo-skeletal system (Blows, Bark; Bone pain, Bark; Rheumatism, Bark); Respiratory system (Cold and flu, Bark)	Roho nësëbi / Rononopa (Ch); Bejuco (Sp)	GOS 23
*Annona montana* Macfad.	CONST: Houses (Frame house, Trunk); HUMFOOD: Food (Edible, Fruit)	Tëto / Tëto chopishi (Ch); Biribá de monte (Sp)	MOV 20
*Annona* sp.	HUMFOOD: Food (Edible, Fruit)	Jënë Biribá (Ch)	CH3
*Cymbopetalum brasiliense* (Vell.) Benth. ex Baill.	HUMFOOD: Food (Edible, Fruit and seeds); MEDVET: Infections and infestations (Leishmaniasis, Root); Skin and subcutaneous tissue (Wounds and cuts, Root); Snakebites and Ray stings (Sankebites, Root)	Bëromo (Ch); Mauro (Sp)	CH4
*Duguetia quitarensis* Benth.	HUMFOOD: Food (Edible, Fruit)	Ahuabaca (Ch)	CH5
*Guatteria discolor* R. E. Fries	CONST: Houses (Frame house, Trunk; To tie house, Bark)	Xahuisi (Ch); Piraquina negra (Sp)	CH6
*Guatteria hyposericea* Diels	CONST: Houses (Muchacho - Ninotí, Trunk; Tie - Xahui, Bark); Thatch (To tie roof, Bark); FUEL: Firewood (Firewood - Caro, Trunk)	Ahuabaca (Ch); Pavo (Sp)	CH7
*Xylopia ligustrifolia* Humb. & Bonpl. ex Dunal	CONST: Houses (Tie - Xahui, Bark; Tirante corto - Cano Bësëcamë, Trunk; To tie house, Bark); Other constructions (Huaracha, Trunk); Thatch (To tie roof, Bark); FUEL: Firewood (Firewood - Caro, Trunk)	Xahuisi (Ch)	MSM 14, SCO 2
*Xylopia peruviana* R.E. Fr.	CONST: Houses (Frame house, Trunk; Hedge - Panë, Trunk; House post- Jibamë, Trunk, Jihuixaca, Trunk, Muchacho - Ninotí, Trunk; Nasëcamëti , Trunk; Pasa ratón - Xoya jabatí, Trunk; Ridgepole - Maracatí, Trunk; Roof beam - Canoxoco, Trunk; Solera - Chitao, Trunk; Tie - Xahui, Bark; Tirante - Cano bëpotó, Trunk; Tirante corto - Cano Bësëcamë, Trunk; Tirante largo - Cano pixquëna, Trunk; To tie fence, Bark; To tie house, Bark); Other constructions (Huaracha, Trunk); Thatch (Techo - Xëhuahacacató, Bark; To tie roof, Bark); FUEL: Firewood (Firewood - Caro, Trunk); HUMFOOD: Food (Edible, Fruit and root); MEDVET: Musculo-skeletal system (Bone pain, Seeds); Skin and subcutaneous tissue (Haemorrhage, Leaf); UTEN&TOOL: Domestic utensils (Basket - Chichabëcasa, Bark; Basket - Chichama, Bark; Basket - Nishicacano, Bark; Basket - Purupachi, Bark; Hammock - Nishi, Bark); Hunting & fishing tools (Arrow - Paca, Trunk); Rope (Rope - Rispichi, Bark)	Tëtëmëtsisi / Xahui (Ch); Piraquina / Pancho (Sp)	MOV 37
*Xylopia polyantha* R.E. Fr.	CONST: Houses (Muchacho - Ninotí, Trunk; Pasa ratón - Xoya jabatí, Trunk; Roof beam - Canoxoco, Trunk; Tirante corto - Cano Bësëcamë, Trunk); Thatch (To tie roof, Bark); FUEL: Firewood (Firewood - Caro, Trunk)	Xahuiria (Ch)	CH8
*Xylopia* sp.	CONST: Houses (Frame house, Trunk; Jihuixaca, Trunk; Muchacho - Ninotí, Trunk; Pasa ratón - Xoya jabatí, Trunk; Ridgepole - Maracatí, Trunk; Roof beam - Canoxoco, Trunk; Tie - Xahui, Bark; Tirante - Cano bëpotó, Trunk; Tirante corto - Cano Bësëcamë, Trunk; Tirante largo - Cano pixquëna, Trunk); FUEL: Firewood (Firewood - Caro, Trunk)	Capëtërëbó (Ch)	ORC 7
Apiaceae			
*Daucus carota* L.	HUMFOOD: Food (Edible, Root); MEDVET: Sensory system (Inflammation of eyes, Root)	Zanahoria (Sp)	
Apocynaceae			
*Aspidosperma excelsum* Benth.	FUEL: Firewood (Firewood - Caro, Trunk)	Tiorcorihua (Ch)	SCO 37
*Aspidosperma megalocarpon* Müll. Arg.	FUEL: Firewood (Firewood - Caro, Trunk); HUMFOOD: Food (Edible, Fruit); MEDVET: Endocrine system (Gallbladder, Bark); Infections and infestations (Malaria and fever, Bark and root); Respiratory system (Cold and flu, Bark and seeds); Skin and subcutaneous tissue (Caracha, Bark); UTEN&TOOL: Domestic utensils (Basket - Chichama, Bark; Pestle of Tacu, Trunk; Tacú - Arusa timatí, Trunk); Labour tools (Axe - Maquë poroma, Trunk; Planting stick - Xësati, Trunk)	Poroma Jihui / Cháchama (Ch); Gabetillo (Sp)	CH9
*Geissospermum reticulatum* (Jacq.) K. Schum.	MEDVET: Infections and infestations (Malaria and fever, Bark)	Jihui Moca (Ch)	GOS 22, SCO 20
*Hancornia speciosa* Gomes	HUMFOOD: Food (Edible, Fruit); MEDVET: Endocrine system (Liver, Bark)	Xabá motoha (Ch); Magaba (Sp)	GOS 16, RBU 17833
*Himatanthus sucuuba* (Spruce ex Müll. Arg.) Woodson	MEDVET: Cultural diseases and disorders (Bad air and scare - Ratëaina, Trunk); Digestive system (Appendicitis, Bark, and exhudate; Diarrhea); Endocrine system (Gallbladder, Exudate; Pancreas, Exudate); General Ailments with Unspecific Symptoms (Body pain, Bark and exhudate; Chest pain, Bark; Headache, Bark); Infections and infestations (Anthelmintic, Bark; Boro, Exudate; Malaria and fever, Exudate); Musculo-skeletal system (Blows, Exudate; Fractures, Bark; Hernia, Exudate; Rheumatism, Leaf)	Jihui bëpiya (Ch); Sucuba (Sp)	DOA 2, 52, ESR 19, GOS 8, 56, MOV 14
*Tabernaemontana linkii* A. DC.	MEDVET: Infections and infestations (Malaria and fever, Bark, leaf and root)	Bahua Quëxti (Ch)	MOV 54, RBU 17863
*Woytkowskia spermatochorda* Woodson	CULT: Personal adornment (Ornament - Maxëití, Seeds; Ornament - Mënëxëtí, Seeds; Ornament - Shinoxëta, Seeds); FUEL: Firewood (Firewood - Caro, Trunk)	Quisi / Chishinicato (Ch)	CH10
Araceae			
*Anthurium* sp.	MEDVET: Digestive system (Appendicitis, Trunk)	Buca Pëhi (Ch)	JSM 52
Philodendron bipinnatifidum SCOtt	CONST: Houses (Tirante largo - Cano pixquëna, Trunk); UTEN&TOOL: Domestic utensils (Basket - Chichama, Trunk)	Guembé (Sp)	CH11
*Philodendron quinquelobum* K. Krause	CONST: Houses (Frame house, Trunk)	Oi Nihi (Ch)	CH12
Xanthosoma sagittifolium L. Scott	HUMFOOD: Food (Edible, Root)	Xëca (Ch); Huaylusa (Sp)	CH13
Xanthosoma striolatum Mart. ex Scott	HUMFOOD: Beberages (Beberage - Chicha, Root); Food (Edible, Root); MEDVET: Skin and subcutaneous tissue (Caracha, Trunk)	Mataca (Ch); Huaylusa / Huaylusa amarilla (Sp)	CH14
Araliaceae			
*Schefflera morototoni* (Aubl.) Maguire, Steyerm. & Frodin	FUEL: Firewood (Firewood - Caro, Root and trunk); MEDVET: Dental health (Toothache, Bark and exhudate); Sensory system (Earache, Bark); Skin and subcutaneous tissue (Wounds and cuts, Bark); UTEN&TOOL: Domestic utensils (Pestle of Tacu, Trunk)	Manihua Jihui / Nahuabëxë (Ch); Guitarrero / Batahua (Sp)	JSM 38, MOV 52
Arecaceae			
*Acrocomia aculeata* (Jacq.) Lodd. ex Mart.	HUMFOOD: Food (Edible, Fruit); UTEN&TOOL: Hunting & fishing tools (Bow - Canatí, Trunk)	Totaí (Ch); Totaí (Sp)	
*Allagoptera leucocalyx* (Drude) Kuntze	MEDVET: Digestive system (Ulcers, Root)	Xahuë jina (Ch); Palmera de la sabana (Sp)	MOV 7
*Astrocaryum aculeatum* G. Mey.	ANIMFOOD: Fodder (Edible, Fruit and seeds); CONST: Houses (Frame house, Trunk; House post- Jibamë, Trunk; Muchacho - Ninotí, Trunk; Tirante - Cano bëpotó, Trunk); Other constructions (Huaracha, Trunk); Thatch (Roof - Xëhuahacacató, Leaf and trunk); CULT: Personal adornment (Ear piercing, Spine; Ornament - Amënoxëta, Spine; Ornament - Anillo, Fruit; Ornament - Rësëti, Spine; To drill seeds, Spine); HUMFOOD: Food (Edible, Palm heart; Edible, Fruit); MEDVET: General Ailments with Unspecific Symptoms (Chest pain, Seeds); Infections and infestations (To get nigua, Spine); Respiratory system (Cold and flu, Seeds; Cough, Seeds); UTEN&TOOL: Domestic utensils (Basket - Bano, Leaf; Basket - Chichabëcasa, Young leaf; Basket - Chichama, Young leaf; Basket - Nishicacano, Young leaf; Basket - Purupachi, Young leaf; Fan - Huëquëti, Young leaf; Hammock - Nishi, Young leaf; Knife, Trunk; Man basket, Young leaf; Mat, Leaf); Hunting & fishing tools (Arrow - Bicobi, Trunk; Arrow - Notsi, Trunk; Arrow - Quërëquë, Trunk; Arrow - Quëspini, Trunk; Arrow - Tahua Quëspini, Trunk; Arrow - Tiopi, Trunk; Arrow, Trunk; Bow - Canatí, Trunk); Labour tools (Axe - Maquë poroma, Trunk; Machete handle, Trunk); Rope (Rope - Rispichi, Young leaf)	Huana / Panimá (Sp)	DOA 40, RBU 17850, ESR 13
*Astrocaryum ulei* Burret	CONST: Houses (Hedge - Panë, Trunk); HUMFOOD: Food (Edible, Fruit); UTEN&TOOL: Domestic utensils (Basket - Chichama, Young leaf; Fan - Huëquëti, Young leaf)	Pani (Ch); Chonta loro / Chonta Tara (Sp)	CH12
*Attalea butyracea* (Mutis ex L.f.) Wess. Boer	CONST: Thatch (Roof - Xëhuahacacató, Leaf); UTEN&TOOL: Domestic utensils (Tacú - Arusa timatí, Trunk); Labour tools (Axe - Maquë poroma, Trunk; Machete handle, Trunk; Planting stick - Xësati, Trunk)	Palla (Sp)	CH13
*Attalea maripa* (Aubl.) Mart.	CONST: Houses (Frame house, Leaf; Ridgepole - Maracatí, Trunk; Tie - Xahui, Young leaf; Tirante - Cano bëpotó, Leaf; To tie house, Young leaf); Thatch (Huaracha roof, Leaf; Ridgepole - Xobomapatí, Leaf; Roof - Xëhuahacacató, Leaf; To tie roof, Young leaf); CULT: Recreational (Toys, Trunk); FUEL: Other fuel (Ceramics - Chomo, Leaf); HUMFOOD: Food (Edible, Palm heart and fruit); MEDVET: Digestive system (Diarrhea); UTEN&TOOL: Domestic utensils (Basket - Bano, Leaf; Basket - Chichama, Young leaf; Basket - Nishicacano, Young leaf; Basket - Purupachi, Young leaf; Basket - Yamachi, Young leaf; Fan - Huëquëti, Young leaf; Knife, Trunk; Mat, Leaf; Pestle of Batan - Chapi, Leaf); Hunting & fishing tools (Arrow - Quëspini, Trunk; Arrow - Tahua Quëspini, Trunk); Labour tools (Axe - Maquë poroma, Trunk; Machete handle, Trunk; Shovel, Trunk); Rope (Rope - Rispichi, Young leaf)	Xëbi choqui (Ch); Motacusillo (Sp)	CH14
*Attalea phalerata* Mart. ex Spreng	CONST: Houses (Frame house, Trunk; Ridgepole - Maracatí, Trunk; Roof beam - Canoxoco, Young leaf; Tie - Xahui, Young leaf; To tie house, Young leaf); Thatch (Huaracha roof, Leaf; Ridgepole - Xobomapatí, Leaf; Roof - Xëhuahacacató, Leaf; To tie roof, Young leaf); CULT: Clothes & accessories (Dress - Moro, Young leaf); Ritual (Fragile children, Root); FUEL: Other fuel (Ceramics - Chomo, Leaf); HUMFOOD: Food (Edible, Fruit); MEDVET: Blood and Cardio-vascular system (Anemia, Root); Dental health (Toothache, Seeds); Digestive system (Diarrhea, Fruit); Infections and infestations (Amoebas, Root; Anthelmintic, Root); Metabolic system and nutrition (Vitamin, Root); Sensory system (Earache, Seeds); Urinary system (Kidneys, Root); UTEN&TOOL: Domestic utensils (Asalle, Young leaf; Basket - Bano, Leaf; Basket - Chichabëcasa, Young leaf; Basket - Chichama, Young leaf; Basket - Nishicacano, Young leaf; Basket - Purupachi, Young leaf; Basket - Yamachi, Young leaf; Batán - Xaxo, Trunk; Fan - Huëquëti, Young leaf; Knife, Trunk; Mat, Leaf); Hunting & fishing tools (Arrow - Quëspini, Trunk; Fishing bait, Seeds); Labour tools (Axe - Maquë poroma, Trunk; Machete handle, Trunk; Planting stick - Xësati, Trunk); Rope (Rope - Rispichi, Young leaf)	Xëbine (Ch); Motacú (Sp)	CH15
*Attalea speciosa* Mart. ex Spreng.	CONST: Houses (Frame house, Trunk; Tirante - Cano bëpotó, Leaf); Thatch (Ridgepole - Xobomapatí, Leaf; Roof - Xëhuahacacató, Leaf); HUMFOOD: Food (Edible, Fruit); MEDVET: Infections and infestations (Leishmaniasis, Seeds); Musculo-skeletal system (Bone pain, Seeds); Skin and subcutaneous tissue (Puchichi, Seeds)	Xëbinihua / Xëni (Ch); Cusi (Sp)	CH16
*Bactris acanthocarpa* Mart.	CONST: Thatch (Huaracha roof, Trunk); HUMFOOD: Food (Edible, Fruit)	Canahuanima (Ch)	CH17
*Bactris gasipaes* Kunth.	CULT: Cosmetic (Hair oil, Fruit); HUMFOOD: Food (Edible, Fruit); UTEN&TOOL: Domestic utensils (Basket - Chichama, Young leaf; Bow to clean cotton, Trunk; Hammock - Nishi, Young leaf; Knife, Trunk; Needle, Spine; Spinning wheel - Ihui bëro, Trunk; Spinning wheel - Ihui, Trunk; Tacú - Arusa timatí, Trunk); Hunting & fishing tools (Arrow - Bicobi, Trunk; Arrow - Notsi, Trunk; Arrow - Paca, Trunk; Arrow - Pio, Trunk; Arrow - Quërëquë, Trunk; Arrow - Quëspini, Trunk; Arrow - Tahua Quëspini, Trunk; Arrow - Tiopi, Trunk; Arrow - Xeña, Trunk; Arrow, Trunk; Bow - Canatí, Trunk); Labour tools (Rake, Leaf; Shovel, Trunk)	Huanima (Ch); Chima (Sp)	DOA46
*Bactris gasipaes* var. *chichagui* (H. Karst.) A.J. Hend.	HUMFOOD: Food (Edible, Fruit); MEDVET: Sensory system (Inflammation of eyes, Palm heart); UTEN&TOOL: Domestic utensils (Basket - Chichabëcasa, Young leaf; Basket - Chichama, Young leaf; Hunting & fishing tools; Bow - Canatí, Trunk); Labour tools (Axe - Maquë poroma, Trunk)	Huanima / Huanima Chitahë / Huanimahua (Ch); Chonta / Chontilla / Pupuña (Sp)	MOV 16, NPZ 8825
*Chelyocarpus chuco* (Mart.) H.E. Moore	CONST: Thatch (Roof - Xëhuahacacató, Young leaf); UTEN&TOOL: Domestic utensils (Basket - Chichama, Young leaf; Fan - Huëquëti, Young leaf; Knife, Petiole); Labour tools (Axe - Maquë poroma, Young leaf; Machete handle, Young leaf)	Hoja Redonda (Sp)	CH18
*Cocos nucifera* L.	HUMFOOD: Food (Edible, Fruit); MEDVET: Skin and subcutaneous tissue (Haemorrhage, Seeds)	Coco (Sp)	Ch19
*Euterpe oleracea* Mart.	CONST: Thatch (Roof - Xëhuahacacató, Leaf); UTEN&TOOL: Domestic utensils (Basket - Bano, Leaf)	Pananë (Ch); Asaí / Palmito (Sp)	CH20
*Euterpe precatoria* Mart.	CONST: Houses (Frame house, Trunk; Hedge - Panë, Trunk; Ridgepole - Maracatí, Trunk); Other constructions (Huaracha, Trunk; Food); Thatch (Huaracha roof, Leaf; Roof - Xëhuahacacató, Leaf); CULT: Personal adornment (Ornament - Maxëití, Fruit; Ornament - Shinoxëta, Seeds); Ritual (Fragile children, Root); FUEL: Other fuel (Ceramics - Chomo, Leaf); HUMFOOD: Beberages (Beberage - Milk, Fruit); Food (Edible, Palm heart, fruit and young leaf); MEDVET: Blood and Cardio-vascular system (Anemia, Root); General Ailments with Unspecific Symptoms (Weakness, Root); Infections and infestations (Amoebas, Root); Insect and athropod bites (Buna bite, Young leaf); Metabolic system and nutrition (Vitamin, Root); Not specified at all (Not specified, Root); Skin and subcutaneous tissue (Caracha, Leaf); Snakebites and Ray stings (Sankebites, Fruit, palm heart, root and trunk); Urinary system (Kidneys, Root); UTEN&TOOL: Domestic utensils (Basket - Bano, Leaf; Basket - Chichama, Young leaf; Basket - Nishicacano, Leaf; Basket - Purupachi, Young leaf; Basket - Yamachi, Leaf; Batán - Xaxo, Trunk; Fan - Huëquëti, Young leaf; Hammock - Nishi, Young leaf; Knife, Trunk; Pestle of Batan - Chapi, Leaf); Hunting & fishing tools (Bow - Canatí, Trunk); Labour tools (Axe - Maquë poroma, Trunk; Hoe, Trunk; Machete handle, Trunk; Planting stick - Xësati, Trunk; Shovel, Trunk); Rope (Rope - Rispichi, Young leaf)	Jënë / Pananë (Ch); Asaí / Palmito (Sp)	ESR 9
*Geonoma deversa* (Poit.) Kunth	CONST: Thatch (Roof - Xëhuahacacató, Leaf)	Jatata (Sp)	CH21
*Geonoma juruana* Dammer	CONST: Houses (Hedge - Panë, Trunk); Other constructions (Huaracha, Trunk); Thatch (Roof - Xëhuahacacató, Leaf); HUMFOOD: Food (Edible, Fruit)	Panë (Ch)	CH22
*Geonoma macrostachys* Mart.	CONST: Thatch (Roof - Xëhuahacacató, Leaf); FUEL: Firewood (Firewood - Caro, Trunk); HUMFOOD: Food (Edible, Fruit); UTEN&TOOL: Hunting & fishing tools (Weapons, Trunk)	Shinibi (Ch)	JSM 24, MOV 27
*Mauritia flexuosa* L. f.	CONST: Thatch (Roof - Xëhuahacacató, Leaf); HUMFOOD: Food (Edible, Fruit); UTEN&TOOL: Domestic utensils (Basket - Chichama, Young leaf; Sieve - Toahi, Young leaf); Labour tools (Axe - Maquë poroma, Trunk; Machete handle, Trunk)	Palma Real (Sp)	CH22
*Oenocarpus balickii* F. Kahn	HUMFOOD: Food (Edible, Fruit)	Xoquëitsama (Ch); Majillo del Tucán (Sp)	RBU 17835
*Oenocarpus bataua* Mart.	CONST: Houses (Ridgepole - Maracatí, Trunk); Other constructions (Huaracha, Trunk); Thatch (Ridgepole - Xobomapatí, Leaf; Roof - Xëhuahacacató, Leaf); CULT: Cosmetic (Hair oil, Fruit); Personal adornment (Ornament - Maxëití, Seeds; Ornament - Mënëxëtí, Seeds; Ornament - Shinoxëta, Seeds); HUMFOOD: Beberages (Beberage - Chicha, Fruit; Beberage - Milk, Fruit); Food (Edible - Larvae, Trunk - Larvae; Edible, Fruit); MEDVET: Endocrine system (Gallbladder, Trunk - Larvae); General Ailments with Unspecific Symptoms (Chest pain, Trunk - Larvae); Infections and infestations (Leishmaniasis, Fruit); Respiratory system (Bronchitis, Trunk - Larvae); Sensory system (Earache, Fruit and trunk larvae); Skin and subcutaneous tissue (Puchichi, Fruit); Snakebites and Ray stings (Sankebites, Fruit); UTEN&TOOL: Domestic utensils (Basket - Bano, Leaf; Basket - Chichama, Young leaf; Basket - Purupachi, Young leaf)	Itsama (Ch); Majo (Sp)	CH23
*Oenocarpus mapora* H. Karst	CONST: Houses (Muchacho - Ninotí, Trunk; Ridgepole - Maracatí, Trunk); HUMFOOD: Beberages (Beberage - Milk, Fruit); Food (Edible - Larvae, Trunk; Edible, Fruit); MEDVET: Snakebites and Ray stings (Sankebites, Trunk - Larvae); UTEN&TOOL: Domestic utensils (Basket - Bano, Leaf; Basket - Purupachi, Young leaf; Tacú - Arusa timatí, Trunk); Hunting & fishing tools (Arrow - Quëspini, Trunk; Bow - Canatí, Trunk)	Quëbo itsama (Ch); Majillo (Sp)	CH24
*Socratea exorrhiza* (Mart.) H. Wendl.	CONST: Houses (Ceiling roof, Trunk; Frame house, Trunk; Hedge - Panë, Trunk; Jaxca Jaxca, Trunk; Tie - Xahui, Young leaf; Tirante largo - Cano pixquëna, Trunk); Other constructions (Chapapa, Trunk; Chicken coop, Trunk; Huaracha, Trunk; Shelves, Trunk; Store corn, Trunk); Thatch (Roof - Xëhuahacacató, Leaf; Roof - Xëhuahacacató, Trunk); HUMFOOD: Food (Edible, Fruit); UTEN&TOOL: Domestic utensils (Basket - Purupachi, Young leaf; Cassava grater, Root; Table, Trunk); Hunting & fishing tools (Bow - Canatí, Trunk)	Onipa (Ch); Pachuba (Sp)	DOA 34
Aristolochiaceae			
*Aristolochia* sp.	MEDVET: Digestive system (Appendicitis, Bark; Diarrhea, Trunk; Stomach ache, Bark); Endocrine system (Liver pain, Bark); General Ailments with Unspecific Symptoms (Headache, Bark; Vomit, Bark)	Maca Huatiapi (Ch)	SCO 32
Asteraceae			
*Vernonia* sp.	MEDVET: General Ailments with Unspecific Symptoms (Inflammation, Trunk)	Cola de caballo (Sp)	DOA 45
*Wulffia baccata* (L.) Kuntze.	MEDVET: Respiratory system (Cold and flu, Leaf)	Bahua Rëxa (Ch)	DOA 29
Bignoniaceae			
*Arrabidaea brachypoda* Bureau	CULT: Personal adornment (Ornament - Shinoxëta, Seeds); MEDVET: Digestive system (Diarrhea, Bark; Stomach ache, Bark); General Ailments with Unspecific Symptoms (Headache, Bark; Vomit, Bark); UTEN&TOOL: Domestic utensils (Basket - Nishicacano, Trunk)	Corama / Nishi Raoxo (Ch); Paquío (Sp)	ESR 1, GOS 7, JSM 16, RBU 17866
*Arrabidaea platyphylla* DC.	MEDVET: Infections and infestations (Boro, Root; Malaria and fever, Root)	Bahua Quëxti (Ch)	CH25
*Arrabidaea* sp.	MEDVET: Sensory system (Inflammation of eyes, Root)	Yoquira (Ch)	CH26
*Ceratophytum tetragonolobum* (Jacq.) Sprague & Sandwith	MEDVET: Cultural diseases and disorders (Bad air and scare - Ratëaina, Leaf); General Ailments with Unspecific Symptoms (Body pain, Leaf; Body pain, Trunk; Headache, Leaf; Vomit, Whole plant ); Infections and infestations (Malaria and fever, Trunk); Musculo-skeletal system (Rheumatism, Leaf)	Boá Nishi / Boá / Bua (Ch); Ajo del monte / Bejuco (Sp)	MOA 4, MOV 65
*Clytostoma* sp.	CONST: Houses (Tie - Xahui, Bark; To tie house, Bark); UTEN&TOOL: Domestic utensils (Basket - Chichama, Bark; Basket - Nishicacano, Bark)	Shino joxotaë / Shino yáquishi (Ch)	CH26
*Crescentia cujete* L.	UTEN&TOOL: Domestic utensils (Container, Fruit)	Tutuma (Sp)	
*Jacaranda copaia* (Aubl.) D. Don.	FUEL: Firewood (Firewood - Caro, Trunk; Firewood, Trunk); Other fuel (Ceramics - Paítí, Bark); MEDVET: Infections and infestations (Scabies, Leaf); Musculo-skeletal system (Ankle pain; Hip pain ); Not specified at all; Respiratory system (Cold and flu); Skin and subcutaneous tissue (Caracha; Wounds and cuts, Leaf)	Pitsopi (Ch)	DOA 20, GOS 35, MOV 49
*Jacaranda obtusifolia* Bonpl.	FUEL: Firewood (Firewood - Caro, Trunk); Other fuel (Ceramics - Tiesto - Pitëxti, Bark); MEDVET: Skin and subcutaneous tissue (Caracha, Bark)	Pitsopi (Ch)	DOA 53, GOS 59
*Mussatia hyacinthina* (Standl.) Sandw.	CONST: Houses (Tie - Xahui, Bark); MEDVET: Digestive system (Diarrhea, Bark; Stomach ache, Bark and leaf); General Ailments with Unspecific Symptoms (Headache, Bark ); Musculo-skeletal system (Bone pain, Bark); Urinary system (Kidneys, Bark)	Xoquë Rapotó (Ch); Chamairo / Chamairo negro (Sp)	CH27
*Pyrostegia dichotoma* Miers ex K. Schum.	MEDVET: General Ailments with Unspecific Symptoms (Vomit, Whole plant)	Nana Nihi (Ch)	CH28
*Tabebuia chrysantha* (Jacq.) Nicholson	CONST: Houses (House post- Jibamë, Trunk)	Tajibo amarillo (Sp)	CH29
*Tabebuia ochracea* (Cham.) Standl.	CONST: Houses (Frame house, Trunk; House post- Jibamë, Trunk; Muchacho - Ninotí, Trunk; Tirante - Cano bëpotó, Trunk); Thatch (Roof - Xëhuahacacató, Trunk); MEDVET: Infections and infestations (Scabies); Skin and subcutaneous tissue (Blisters ); Urinary system (Kidneys); UTEN&TOOL: Domestic utensils (Gavel to make dress, Trunk; Pestle of Tacu, Trunk)	Nishó (Ch); Tajibo negro (Sp)	GOS 4
*Tabebuia serratifolia* (Vahl) G. Nicholson	CONST: Houses (Frame house, Trunk; House post- Jibamë, Trunk); HUMFOOD: Food (Edible, Trunk); UTEN&TOOL: Domestic utensils (Batán - Xaxo, Trunk; Tacú - Arusa timatí, Trunk)	Tajibo (Sp)	CH30
*Tabebuia* sp.	CONST: Houses (Frame house, Trunk; Muchacho - Ninotí, Trunk; Pasa ratón - Xoya jabatí, Trunk; Solera - Chitao, Trunk; Tie - Xahui, Bark; Tirante - Cano bëpotó, Trunk; Tirante largo - Cano pixquëna, Trunk); FUEL: Firewood (Firewood - Caro, Trunk); HUMFOOD: Food (Edible, Fruit); MEDVET: Endocrine system (Gallbladder, Root, seeds and trunk; Liver pain, Bark, root and seeds); Sensory system (Inflammation of eyes, Bark, leaf, and root; Conjunctivitis, Root; Earache, Root)	Toromuca (Ch); Yuquilla (Sp)	MSM 6, 9
Bixaceae			
*Bixa orellana* L.	CULT: Personal adornment (Ornament - Maxëití, Seeds; Ornament - Xapo, Seeds); HUMFOOD: Food (Edible, Seeds); MEDVET: Blood and Cardio-vascular system (Heartache); Cultural diseases and disorders (Evil eye, Trunk); Digestive system (Stomach ache, Young leaf); Endocrine system (Liver pain, Young leaf); General Ailments with Unspecific Symptoms (Headache, Leaf; Inflammation ); Infections and infestations (Leishmaniasis, Young leaf; Malaria and fever, Exudate and leaf); Respiratory system (Cold and flu); Sensory system (Inflammation of eyes, Exudate and seeds); Skin and subcutaneous tissue (Haemorrhage, Root and young leaf; Puchichi, Young leaf); Urinary system (Kidneys)	Maxë (Ch); Urucú (Sp)	GOS 13
Boraginaceae			
*Cordia alliodora* (Ruiz. & Pav.) Oken.	CONST: Houses (Frame house, Trunk)	Pabo (Ch)	JSM 7, 44
*Cordia ucayaliensis* (I.M. Johnst.) I.M. Johnst.	CONST: Houses (To tie house, Bark); FUEL: Firewood (Firewood - Caro, Trunk)	Pabo (Ch)	MOV 33, SCO 36
Bromeliaceae			
*Ananas comosum* (L.) Merr.	CULT: Ritual (Fragile children, Leaf); HUMFOOD: Food (Edible, Fruit); MEDVET: Digestive system (Diarrhea, Leaf); General Ailments with Unspecific Symptoms (Vomit, Fruit)	Piña (Sp)	
Indet. sp. 1	HUMFOOD: Food (Edible, Fruit); MEDVET: Cultural diseases and disorders (Bad air and scare - Ratëaina, Seeds)	Xabá cacatao / Xabá Camano (Ch)	MOV 4
Burseraceae			
*Protium aracouchini* (Aubl.) Marchand	MEDVET: Cultural diseases and disorders (Difficulty speaking, Leaf)	Toshi xeta (Ch)	DOA 10
*Protium sagotianum* Marchand	FUEL: Firewood (Firewood - Caro, Trunk); HUMFOOD: Food (Edible, Fruit)	Bëisiti Corihua (Ch); Isigo (Sp)	CH31
Cannabaceae			
*Celtis schippii* Standl.	FUEL: Firewood (Firewood - Caro, Trunk)	Cátsisi (Ch)	CH32
*Trema micrantha* (L.) Blume	ANIMFOOD: Fodder (Edible, Fruit); FUEL: Firewood (Firewood - Caro, Trunk); HUMFOOD: Food (Edible, Fruit)	Merabí (Ch)	JSM 61
Capparaceae			
*Capparis coimbrana* Cornejo & Iltis	FUEL: Firewood (Firewood - Caro, Trunk); HUMFOOD: Food (Edible, Fruit); MEDVET: Endocrine system (Gallbladder, Bark); Infections and infestations (Malaria and fever, Bark)	Maca (Ch)	GOS 1, MOV 24
Caricaceae			
*Carica papaya* L.	HUMFOOD: Food (Edible, Fruit); MEDVET: Dental health (Toothache, Root); Digestive system (Diarrhea, Exudate); Infections and infestations (Amoebas, Leaf and seeds; Anthelmintic, Leaf and seeds); Insect and athropod bites (Insectbite, Seeds); Respiratory system (Cold and flu, Exudate ); Sensory system (Earache, Exudate); Snakebites and Ray stings (Sankebites, Root)	Mapayo (Ch); Papaya (Sp)	GCM 10
*Jacaratia digitata* (Poepp. & Endl.) Solms	HUMFOOD: Food (Edible, Fruit); MEDVET: Dental health (Toothache, Root); General Ailments with Unspecific Symptoms (Body pain, Trunk; Headache, Leaf); Musculo-skeletal system (Bone pain, Trunk); Not specified at all (Not specified, Fruit); Snakebites and Ray stings (Sankebites, Root)	Cana bohi / Huaquibí / Mapayo bërë (Ch); Papaya del monte / papaya macho (Sp)	GOS 31
Caryocaraceae			
*Caryocar dentatum* Gleason	ANIMFOOD: Fodder (Edible, Fruit); CULT: Clothes & accessories (Wash dress, Bark); Personal adornment (Ornament - Mënëxëtí, Bark); MEDVET: Not specified at all (Not specified)	Jënë carama (Ch)	DOA 57, GOS 54
Celastraceae			
*Salacia elliptica* (Mart. ex Schult.) G. Don.	FUEL: Firewood (Firewood - Caro, Trunk); HUMFOOD: Food (Edible, Fruit); MEDVET: Digestive system (Diarrhea, Leaf); Urinary system (Kidneys, Root)	Nëxo quërimati (Ch); Huapamú (Sp)	DOA 47, GOS 42, MOA 3
*Salacia gigantea* Loes	CONST: Houses (Tie - Xahui, Bark); HUMFOOD: Food (Edible, Fruit)	Ahuax Xtëpoco (Ch)	CH33
*Tontelea ovalifolia* subsp. *richardii* (Peyr.) Görts & Mennega	HUMFOOD: Food (Edible, Fruit); MEDVET: Endocrine system (Liver pain, Fruit)	Quërë matí (Ch); Guapomó (Sp)	CH34
Chrysobalanaceae			
*Hirtella gracilipes* (Hook. f.) Prance	MEDVET: Skin and subcutaneous tissue (Caracha, Bark)	Xaba chana (Ch)	MSM 4, RBU 17832
*Hirtella pilosissima* Mart. & Zucc.	CONST: Houses (House post- Jibamë, Trunk); CULT: Clothes & accessories (Dress - Moro, Bark); FUEL: Firewood (Firewood - Caro, Trunk); HUMFOOD: Food (Edible, Fruit); MEDVET: Musculo-skeletal system (Fractures, Bark); Skin and subcutaneous tissue (Caracha, Bark); UTEN&TOOL: Domestic utensils (Gavel to make dress, Trunk; Pestle of Tacu, Trunk; Tacú - Arusa timatí, Trunk); Labour tools (Machete handle, Trunk; Planting stick - Xësati, Trunk; Spade handle, Trunk)	Chanahua / Moro choxtí (Ch); Coloradillo / Cuchi (Sp)	CH34
*Licania intrapetiolaris* Spruce ex. Hook f.	CONST: Houses (Frame house, Trunk; Hedge - Panë, Trunk; House post- Jibamë, Trunk; Jihuixaca, Trunk; Manipoatí, Trunk; Muchacho - Ninotí, Trunk; Pasa ratón - Xoya jabatí, Trunk; Ridgepole - Maracatí, Trunk; Roof beam - Canoxoco, Trunk; Sabrillo, Trunk; Solera - Chitao, Trunk; Tirante largo - Cano pixquëna, Trunk); Other constructions (Huaracha, Trunk); FUEL: Firewood (Firewood - Caro, Trunk); Other fuel (Ceramics - Chomo, Bark; Ceramics - Comëno, Bark; Ceramics - Paítí, Bark); HUMFOOD: Food (Edible, Fruit); MEDVET: General Ailments with Unspecific Symptoms (Chest pain, Seeds); Insect and athropod bites (Insectbite, Bark); Musculo-skeletal system (Bone pain, Seeds); UTEN&TOOL: Domestic utensils (Pestle of Tacu, Trunk)	Shishaxë (Ch); Cacharí (Sp)	CH35
*Licania octandra* subsp. *pallida* (Hook. f.) Prance	CONST: Houses (Hedge - Panë, Trunk; Xano, Trunk); FUEL: Firewood (Firewood - Caro, Trunk); Other fuel (Ceramics - Chomo, Bark; Ceramics - Comëno, Bark; Ceramics - Paítí, Bark; Ceramics - Tiesto - Pitëxti, Bark); HUMFOOD: Food (Edible, Fruit); UTEN&TOOL: Domestic utensils (Basket - Chichabëcasa, Bark; Basket - Chichama, Bark; Batán - Xaxo, Trunk)	Mëhi (Ch); Caripe (Sp)	MSM 10
Clusiaceae			
*Garcinia madruno* (Kunth) Hammel	ANIMFOOD: Fodder (Edible, Fruit); HUMFOOD: Food (Edible, Fruit); Food aditives (Additive coca chewing, Fruit); MEDVET: Digestive system (Diarrhea, Bark; Stomach ache, Bark); Skin and subcutaneous tissue (Puchichi, Young leaf)	Shiquishi / Tahuatë (Ch); Achachairú (Sp)	DOA 12, JSM 33
*Tovomita* sp.	FUEL: Firewood (Firewood - Caro, Trunk)	Áhuara Macha (Ch)	MOV 28
Indet. sp. 1	CONST: Houses (Frame house, Trunk; Hedge - Panë, Trunk; House post- Jibamë, Trunk; Muchacho - Ninotí, Trunk; Ridgepole - Maracatí, Trunk; Roof beam - Canoxoco, Trunk; Solera - Chitao, Trunk; Tie - Xahui, Bark; Tirante - Cano bëpotó, Trunk; Tirante largo - Cano pixquëna, Trunk); HUMFOOD: Food (Edible, Fruit); UTEN&TOOL: Domestic utensils (Tacú - Arusa timatí, Trunk)	Bacurí (Ch); Motoa (Sp)	CH36
Cochleospermaceae			
*Cochlospermum orinocense* (H.B.K.) Steudel	CONST: Houses (Tie - Xahui, Bark)	Algodoncillo (Sp)	CH37
Combretaceae			
*Terminalia amazonica* (Gmel.) Exell	MEDVET: Skin and subcutaneous tissue (Wounds and cuts, Bark and leaf)	Verdolago (Sp)	CH38
Connaraceae			
*Connarus ruber* (Poepp. & Endl.) Planch.	CULT: Recreational (Toys, Fruit and leaf)	Pitso tapa (Ch)	GCM 6, MSM 15
Convolvulaceae			
*Ipomoea batas* Lam.	HUMFOOD: Beberages (Beberage - Chicha, Root); Food (Edible, Root); MEDVET: Digestive system (Appendicitis, Leaf; Stomach ache, Flower); Endocrine system (Gallbladder, Seeds); General Ailments with Unspecific Symptoms (For bathing sick chlidren, Whole plant; Headache, Root); Infections and infestations (Malaria and fever, Leaf); Respiratory system (Bronchitis, Leaf and root; Cold and flu, Leaf and root)	Cari (Ch); Camote (Sp)	
Costaceae			
*Costus scaber* Ruiz. & Pav.	MEDVET: Blood and Cardio-vascular system (Heartache, Trunk); Cultural diseases and disorders (Bad air and scare - Ratëaina, Root); Dental health (Toothache, Root and seeds); Digestive system (Appendicitis, Whole plant; Diarrhea, Fruit, root, trunk and young leaf ; Stomach ache, Trunk); Endocrine system (Gallbladder, Trunk and whole plant; Gallbladder, Whole plant and root); Pancreas, Trunk); General Ailments with Unspecific Symptoms (Headache, Root; Vomit, Fruit, leaf, root, seeds, trunk and whole plant); Infections and infestations (Hepatitis, Whole plant); Musculo-skeletal system (Swelling, Trunk); Skin and subcutaneous tissue (Burns, Leaf); Urinary system (Kidney infection, Trunk; Kidney pain, Leaf and trunk; Kidneys, Leaf and trunk; Yellow urine, Trunk)	Bushishi (Ch); Cahuasha / Caña / Caña agria (Sp)	DOA 48, GOS 45, MOA 6, SCO 29, JSM 56
Crassulaceae			
*Bryophyllum* sp.	MEDVET: Infections and infestations (Leishmaniasis, Leaf); Musculo-skeletal system (Blows, Leaf; Swelling, Leaf); Skin and subcutaneous tissue (Puchichi, Leaf)	Bai Ati (Ch); Fortuna (Sp)	CH39
Cucurbitaceae			
*Citrullus vulgaris* Schrad.	HUMFOOD: Food (Edible, Fruit); UTEN&TOOL: Domestic utensils (Container, Fruit)	Sania (Ch); Sandía (Sp)	
*Cucumis sativus* L.	HUMFOOD: Food (Edible, Fruit)	Pepino (Sp)	
*Cucurbita moschata* Duchesne	HUMFOOD: Food (Edible, Fruit)	Zapallo (Sp)	
*Cucurbita* sp.	HUMFOOD: Food (Edible, Seeds); MEDVET: Skin and subcutaneous tissue (Caracha, Leaf; Skin fungus, Leaf); UTEN&TOOL: Domestic utensils (Container, Fruit)	Mate / Mate bejuco (Sp)	
Indet. sp. 1	HUMFOOD: Food (Edible, Fruit)	Huaramë (Ch); Joco (Sp)	CH40
Cyperaceae			
*Cyperus* sp.	MEDVET: Digestive system (Appendicitis, Whole plant; Diarrhea, Root and trunk; Stomach ache, Trunk); Endocrine system (Liver pain, Root); General Ailments with Unspecific Symptoms (Inflammation, Leaf; Vomit, Root, seeds and trunk); Infections and infestations (Malaria and fever, Root)	Tsanona (Ch); Cahuasha (Sp)	GOS 41
*Diplasia karatifolia* Rich.	MEDVET: Digestive system (Constipation, Trunk); Pregnancy, birth and puerperial (Birth, Root)	Cortadera (Sp)	ESR 16
Dennstaedtiaceae			
*Pteridium* sp.	MEDVET: Digestive system (Stomach ache, Root); Endocrine system (Gallbladder, Root)	Jasini huitahuo (Ch)	MOV 53
Dichapetalaceae			
*Dichapetalum spruceanum* Baill.	FUEL: Firewood (Firewood - Caro, Trunk)	Nishi cobo (Ch)	MOV 43
Dilleniaceae			
*Curatella americana* L.	FUEL: Firewood (Firewood - Caro, Trunk); MEDVET: Digestive system (Diarrhea); Endocrine system (Liver); General Ailments with Unspecific Symptoms (Chest pain, Bark); Infections and infestations (Malaria and fever, Bark); Respiratory system (Cough, Bark)	Xaba tapahua (Ch)	JSM 9, RBU 17846, GCM 3
*Doliocarpus* sp.	CONST: Houses (Ridgepole - Maracatí, Trunk; Tie - Xahui, Bark; To tie house, Bark); Thatch (To tie roof, Bark); MEDVET: Digestive system (Diarrhea, Bark)	Quëbo xamahua (Ch)	CH41
Dioscoreaceae			
*Dioscorea latifolia* Benth.	HUMFOOD: Beberages (Beberage - Chicha, Root); Food (Edible - Chive, Root; Edible, Root)	Chaxo Poa (Ch); Bachi (Sp)	CH42
Erythroxylaceae			
*Erythroxylum coca* Lam.	FUEL: Firewood (Firewood - Caro, Trunk); MEDVET: Cultural diseases and disorders (Bad air and scare - Ratëaina, Leaf); Digestive system (Stomach ache, Leaf); Endocrine system (Liver pain, Leaf); UTEN&TOOL: Labour tools (Awl, Trunk)	Huara huara (Ch); Coca (Sp)	DOA 8, JSM 28
Euphorbiaceae			
*Alchornea* sp.	CONST: Houses (House post- Jibamë, Trunk; Muchacho - Ninotí, Trunk; Pasa ratón - Xoya jabatí, Trunk; Tirante - Cano bëpotó, Trunk; Tirante corto - Cano Bësëcamë, Trunk; Tirante largo - Cano pixquëna, Trunk); Other constructions (Huaracha, Trunk); CULT: Ritual (Fragile children, Leaf); FUEL: Firewood (Firewood - Caro, Trunk); MEDVET: Skin and subcutaneous tissue (Caracha, Bark)	Manahuita (Ch); Cama (Sp)	CH43
*Cleidion* sp.	ANIMFOOD: Fodder (Edible, Fruit); CONST: Houses (Hedge - Panë, Trunk); FUEL: Firewood (Firewood - Caro, Trunk)	Huacaxapo (Ch)	MOV 45, MSM 13
*Croton lechleri* Müll. Arg.	MEDVET: Skin and subcutaneous tissue (Puchichi, Exudate; Wounds and cuts)	Sangre de Grado (Sp)	CH44
*Croton matourensis* Aublet	CONST: Houses (Hedge - Panë, Trunk; Muchacho - Ninotí, Trunk; Tirante largo - Cano pixquëna, Trunk); Other constructions (Huaracha, Trunk); UTEN&TOOL: Domestic utensils (Batán - Xaxo, Trunk; Cramps, Seeds; Pestle of Batan - Chapi, Trunk; Pestle of Tacu, Trunk; Tacú - Arusa timatí, Trunk)	Aliso (Sp)	CH45
*Croton trinitatis* Millsp.	MEDVET: Veterinary (Distemper)	Taxa Bahueti (Ch); Malvilla (Sp)	DOA 1
*Croton* sp.	MEDVET: Endocrine system (Gallbladder); General Ailments with Unspecific Symptoms (Headache, Leaf); Infections and infestations (Malaria and fever, Leaf); Musculo-skeletal system (Haematoma, Whole plant); Not specified at all (Insomnia in chlidren); Respiratory system (Cold and flu, Whole plant); Sensory system (Inflammation of eyes, Leaf); UTEN&TOOL: Domestic utensils (Broom, Whole plant)	Matsëti (Ch); Malva (Sp)	MOV 64
*Hevea brasiliensis* (Willd. ex A. Juss.) Müll. Arg.	CULT: Clothes & accessories (Rubber shoes, Exudate); Personal adornment (Ornament - Shinoxëta, Seeds); FUEL: Firewood (Firewood - Caro, Trunk); Other fuel (Ceramics - Comëno, Bark); MEDVET: Infections and infestations (Boro, Exudate; Malaria and fever, Leaf); Musculo-skeletal system (Bone pain, Bark); Skin and subcutaneous tissue (Facila blemishes, Bark); SALE: Sale (Siringa, Exudate)	Carama (Ch); Caucho / Siringa (Sp)	CH45
*Jatropha curcas* L.	MEDVET: Infections and infestations (Malaria and fever, Leaf)	Piñon (Sp)	CH46
*Jatropha gossypiifolia* L.	HUMFOOD: Food (Edible, Fruit); MEDVET: Cultural diseases and disorders (Bad air and scare - Ratëaina, Leaf); Insect and athropod bites (Centipede bite, Bark and leaf); Musculo-skeletal system (Bone pain, Seeds; Cramps, Seeds; Fractures, Seeds; Rheumatism, Seeds); Reproductive system and sex health (Contraceptive, Seeds); Respiratory system (Cramps, Seeds and seeds); Skin and subcutaneous tissue (Wounds and cuts, Seeds); Snakebites and Ray stings (Sankebites, Bark and leaf); Urinary system (Kidneys, Bark, leaf and seeds)	Raë (Ch); Copaiba / Piñón morado (Sp)	DOA 43, GCM 9, GOS 51
*Mabea fistulifera* Mart.	ANIMFOOD: Fodder (Edible, Fruit); MEDVET: Infections and infestations (Anthelmintic, Exudate; Boro, Exudate); Insect and athropod bites (Insectbite, Exudate); UTEN&TOOL: Hunting & fishing tools (Barbasco - Axa, Fruit)	Piri (Ch); Leche leche (Sp)	DOA 5, JSM 35
*Manihot esculenta* Crantz	HUMFOOD: Beberages (Beberage - Chicha, Root); Food (Edible - Chive, Root; Edible, Root); MEDVET: Dental health (Toothache, Seeds); Endocrine system (Liver pain, Leaf); Musculo-skeletal system (Swelling, Root); Skin and subcutaneous tissue (Puchichi, Root; Wounds and cuts, Root); Urinary system (Kidney pain, Root; Kidneys, Root )	Atsa / Atsa Chëquë / Atsa Hosho / Atsa Nasisi / Atsa Noa / Atsa Pohi Quinihua / Atsa Raoxo / Atsa Shini / Atsa Tocha / Kanaki / Raox tëtoya / Rono Atsa / Shoshapo / Xëto itsa / Xoya atsa (Ch); Rama blanca / Rama morada / Yuca / Yuca de rama choca o café / Yuca piraquina (Sp)	
*Omphalea diandra* L.	CULT: Personal adornment (Ornament - Chimo, Bark)	Chimo / Jianati (Ch); Bejuco (Sp)	
*Ricinus comunis* L.	CULT: Personal adornment (Ornament - Maxëití, Seeds); MEDVET: Musculo-skeletal system (Bone pain, Leaf; Fractures, Leaf); Respiratory system (Cold and flu, Leaf)	Rarë (Ch); Macororo / Matapalo (Sp)	ESR 22, MOA 2
Fabaceae			
*Acacia loretensis* J.F. Macbr.	CONST: Houses (House post- Jibamë, Trunk; Tie - Xahui, Bark; Tirante - Cano bëpotó, Trunk); FUEL: Firewood (Firewood - Caro, Trunk); Other fuel (Ceramics - Chomo, Bark); MEDVET: Respiratory system (Cough, Bark); Skin and subcutaneous tissue (Caracha, Bark; Empeine, Bark)	Caxcono / Capë Caxcono / Isnëpa (Ch); Cari cari (Sp)	BCM 11, DOA 23
*Acacia* sp.	CONST: Houses (Frame house, Bark); HUMFOOD: Food (Edible, Fruit); MEDVET: Digestive system (Stomach ache, Bark); Endocrine system (Diabetes, Bark; Gallbladder, Bark); Reproductive system and sex health (Vaginal douche, Bark); Respiratory system (Cold and flu, Bark and root); Skin and subcutaneous tissue (Haemorrhage, Bark; Wounds and cuts, Bark )	Sipamë (Ch); Tipa (Sp)	GOS 53
*Amburana cearensis* (Allemão) A.C. Sm.	CONST: Houses (Tie - Xahui, Bark); Other constructions (Huaracha, Trunk); HUMFOOD: Food (Edible, Fruit); MEDVET: Cultural diseases and disorders (Bad air and scare - Ratëaina, Bark and leaf); General Ailments with Unspecific Symptoms (Headache, Bark); Infections and infestations (Malaria and fever, Bark); Skin and subcutaneous tissue (Facila blemishes ); Veterinary (Distemper, Bark ); UTEN&TOOL: Domestic utensils (Batán - Xaxo, Trunk; Pestle of Tacu, Trunk; Spoon, Trunk; Tacú - Arusa timatí, Trunk); Transportation (Canoe, Trunk)	Quixono (Ch); Tumi / Roble (Sp)	CH47
*Apuleia leiocarpa* (Vogel) J.F. Macbr.	CONST: Houses (Frame house, Trunk; Hedge - Panë, Trunk; House post- Jibamë, Trunk; Muchacho - Ninotí, Trunk; Roof beam - Canoxoco, Trunk; Tirante - Cano bëpotó, Trunk; Xano, Trunk); FUEL: Firewood (Firewood - Caro, Trunk); Other fuel (Ceramics - Paítí, Bark); HUMFOOD: Food (Edible, Fruit); MEDVET: Digestive system (Diarrhea, Bark fruit and root); Endocrine system (Liver pain, Leaf); Sensory system (Inflammation of eyes, Bark); Skin and subcutaneous tissue (Haemorrhage, Seeds; Wounds and cuts, Bark and fruit); UTEN&TOOL: Domestic utensils (Batán - Xaxo, Trunk; Pestle of Batan - Chapi, Root; Pestle of Batan - Chapi, Trunk; Pestle of Tacu, Trunk; Tacú - Arusa timatí, Trunk); Labour tools (Planting stick - Xësati, Trunk)	Mani / Mani tapono (Ch); Almendrillo / Amarillo (Sp)	CH48
*Bauhinia guianensis* Aubl.	MEDVET: Cultural diseases and disorders (Bad air and scare - Ratëaina, Root); Digestive system (Diarrhea, Trunk); Endocrine system (Diabetes, Leaf); General Ailments with Unspecific Symptoms (Vomit, Bark and seeds); Infections and infestations (Amoebas, Trunk; Leishmaniasis, Trunk); Musculo-skeletal system (Hip pain, Trunk); Respiratory system (Cold and flu, Trunk); Sensory system (Eyes, Trunk); Urinary system (Kidneys, Trunk)	Nishi isanuma / Nishi para (Ch); Bejuco blana / Pataigue (Sp)	GOS 40, MOV 44, RBU 17855, SCO 17
*Bauhinia* sp.	CULT: Ritual (To make hunting dogs, Leaf); MEDVET: Endocrine system (Diabetes, Trunk)	Camanó pahoqui (Ch)	GOS 52
*Bauhinia straussiana* Harms	CULT: Clothes & accessories (Dress - Moro, Bark); HUMFOOD: Food (Edible, Fruit); MEDVET: Insect and athropod bites (Buna bite, Exudate); Skin and subcutaneous tissue (Burns, Exudate)	Chirimoya (Sp)	GOS 27
Chamaecrista nictitans (L.) Moench	CULT: Ritual (Crying children, Leaf); MEDVET: Cultural diseases and disorders (Bad air and scare - Ratëaina, Bark and leaf)	Oxa nihi (Ch); Dormilón (Sp)	DOA 55, GOS 58
*Deguelia amazonica* Killip	MEDVET: Infections and infestations (Leishmaniasis, Trunk); UTEN&TOOL: Hunting & fishing tools (Barbasco - CapëItsa, Root and trunk)	Capë Itsa (Ch); Barbasco (Sp)	CH49
*Derris amazonica* Killip	UTEN&TOOL: Hunting & fishing tools (Barbasco - Axa, Trunk and root)	Axaria (Ch)	CH50
*Derris floribunda* (Benth.) Ducke	UTEN&TOOL: Hunting & fishing tools (Barbasco - Axa, Bark, leaf and root)	Axa (Ch); Barbasco / Bejuco blanco (Sp)	CH51
*Dipteryx alata* Vogel	MEDVET: Skin and subcutaneous tissue (Caracha, Bark)	Nihi pëpëcho (Ch)	GOS 32
*Dipteryx odorata* (Aubl.) Willd.	CONST: Houses (Frame house, Trunk); CULT: Personal adornment (Ornament - Maxëití, Seeds)	Boë (Ch); Yatorana (Sp)	SCO 1
*Hymenaea courbaril* L.	CONST: Houses (Frame house, Trunk; Solera - Chitao, Trunk); FUEL: Firewood (Firewood - Caro, Trunk); Other fuel (Ceramics - Comëno, Trunk); HUMFOOD: Food (Edible, Fruit); MEDVET: Digestive system (Diarrhea, Bark); General Ailments with Unspecific Symptoms (Body pain, Bark); Infections and infestations (Amoebas, Bark; Malaria and fever, Bark); Musculo-skeletal system (Hip pain, Bark; Rheumatism, Bark); Respiratory system (Cold and flu, Bark and seeds); Veterinary (Distemper, Bark); UTEN&TOOL: Domestic utensils (Batán - Xaxo, Trunk; Mattress, Bark; Pestle of Tacu, Trunk; Tacú - Arusa timatí, Trunk)	Corama / Cura pisi (Ch); Paquío (Sp)	MOV 25
*Inga edulis* Mart.	CONST: Houses (Xano, Trunk); FUEL: Firewood (Firewood - Caro, Trunk); HUMFOOD: Food (Edible, Fruit); UTEN&TOOL: Hunting & fishing tools (Arrow, Trunk)	Xënanë (Ch); Pacai (Sp)	DOA 51, JSM 1
*Inga fagifolia* G. Don.	HUMFOOD: Food (Edible, Fruit)	Ahuapi Xënanë (Ch); Pacai del Monte (Sp)	CH52
*Inga marginata* Willd.	HUMFOOD: Food (Edible, Fruit); MEDVET: Not specified at all (Not specified, Leaf)	Shipi Xënanë (Ch); Pacai (Sp)	GOS 38
*Inga* sp. 1	HUMFOOD: Food (Edible, Fruit)	Roho xënanë (Ch); Pacai (Sp)	CH53
*Inga* sp. 2	MEDVET: Infections and infestations (Anthelmintic, Fruit)	Xënanë (Ch)	GOS 36
*Inga* sp. 3	FUEL: Firewood (Firewood - Caro, Trunk); HUMFOOD: Food (Edible, Fruit)	Comoni (Ch); Pacai (Sp)	CH54
*Inga* sp. 4	CONST: Houses (Tie - Xahui, Bark); Thatch (To tie roof, Bark)	Chërë Xahui (Ch)	CH55
*Inga* sp. 5	CONST: Houses (Muchacho - Ninotí, Trunk; Pasa ratón - Xoya jabatí, Trunk; Roof beam - Canoxoco, Trunk; Solera - Chitao, Trunk)	Chira Xahui (Ch)	CH56
*Inga* sp. 6	HUMFOOD: Food (Edible, Fruit)	Huayhuatia Xënanë (Ch); Pacai (Sp)	CH57
*Inga* sp. 7	HUMFOOD: Food (Edible, Fruit)	Rayo xënanë (Ch)	CH58
*Machaerium acutifolium* Vogel	MEDVET: Infections and infestations (Malaria and fever, Root)	Jihui rashia (Ch)	GOS 37
*Ormosia nobilis* Tul.	ANIMFOOD: Fodder (Edible, Fruit); CULT: Personal adornment (Ornament - Shinoxëta, Seeds); MEDVET: Infections and infestations (Malaria and fever, Bark); Reproductive system and sex health (Menstrual pain, Bark and seeds); Skin and subcutaneous tissue (Haemorrhage, Seeds; Puchichi, Bark)	Tëhuëti / Xëta tarati / Xëta tënëti (Ch); Sirari (Sp)	CH59
*Poeppigia procera* C. Presl.	CONST: Houses (House post- Jibamë, Trunk); Other constructions (Huaracha, Trunk); FUEL: Firewood (Firewood - Caro, Trunk)	Matsa Quití (Ch)	CH60
*Pithocellobium corymbosum* (Rich.) Benth.	CONST: Houses (House post- Jibamë, Trunk; Roof beam - Canoxoco, Trunk); MEDVET: Digestive system (Diarrhea, Bark); Skin and subcutaneous tissue (Wounds and cuts); UTEN&TOOL: Domestic utensils (Batán - Xaxo, Trunk; Pestle of Batan - Chapi, Trunk; Pestle of Tacu, Trunk; Tacú - Arusa timatí, Trunk)	Maní (Sp)	CH61
*Platymiscium stipulare* Benth.	MEDVET: Digestive system (Diarrhea, Leaf); General Ailments with Unspecific Symptoms (Vomit, Leaf); Skin and subcutaneous tissue (Acne, Leaf)	Boë xëni (Ch)	GOS 17
*Samanea tubulosa* (Benth.) Barneby & J.W. Grimes	MEDVET: General Ailments with Unspecific Symptoms (Headache, Bark)		GOS 39
*Sclerolobium radlkoferi* Rusby	MEDVET: Skin and subcutaneous tissue (Caracha, Bark)	Huasi Canó (Ch)	CH62
*Senna herzogii* (Harms) H.S. Irwin & Barneby	CONST: Houses (To tie house, Bark); FUEL: Firewood (Firewood - Caro, Bark)	Pabo (Ch)	JSM 59
*Senna occidentalis* (L.) Link.	HUMFOOD: Food (Edible, Fruit); MEDVET: Digestive system (Diarrhea); General Ailments with Unspecific Symptoms (Vomit, Fruit)	Túsa (Ch)	MOV 1
*Stryphnodendron guianense* (Aubl.) Benth.	FUEL: Firewood (Firewood - Caro, Trunk)	Ihui pisi (Ch); Carachupa (Sp)	MOV 48, SCO 34
*Sweetia fruticosa* Spreng.	CONST: Houses (Frame house, Trunk; House post- Jibamë, Trunk)	Canamashía (Ch)	CH53
*Tamarindus indica* L.	HUMFOOD: Food (Edible, Fruit); MEDVET: Digestive system (Stomach ache, Seeds)	Tamarindo (Sp)	CH54
*Vataireopsis speciosa* Ducke	FUEL: Firewood (Firewood - Caro, Trunk)	Ihui pisi (Ch)	CH55
*Vigna unguiculata* (L.) Walp.	HUMFOOD: Food (Edible, Seeds)	Birijori (Ch); Frejol (Sp)	CH56
*Zornia latifolia* Sm.	CULT: Ritual (Crying children)	Hoxa Nihi (Ch); Mujer Yoxa (Sp)	CH57
Indet. sp. 1	CULT: Ritual (Good luck, Leaf)	Tëtëmabaspá (Ch)	CH58
Flacourtiaceae			
Indet. sp. 1	CONST: Houses (Frame house, Trunk; Jihuixaca, Trunk; Muchacho - Ninotí, Trunk; Pasa ratón - Xoya jabatí, Trunk; Ridgepole - Maracatí, Trunk; Roof beam - Canoxoco, Trunk; Solera - Chitao, Trunk; Tirante - Cano bëpotó, Trunk; Tirante largo - Cano pixquëna, Trunk; Xano, Trunk); Thatch (Roof - Xëhuahacacató, Bark); UTEN&TOOL: Domestic utensils (Batán - Xaxo, Trunk)	Xaxo atí (Ch); Canelon (Sp)	CH59
Gesneriaceae			
*Codonanthe calcarata* (Miq.) Hanst.	MEDVET: Digestive system (Diarrhea, Trunk)	Chixopa (Ch)	CH60
Heliconiaceae			
*Heliconia hirsuta* L.f.	UTEN&TOOL: Domestic utensils (Pestle of Tacu, Trunk)	Tsacahuico (Ch)	RBU 17852, SCO 11
*Heliconia* sp.	CONST: Thatch (Roof - Xëhuahacacató, Leaf); MEDVET: Digestive system (Diarrhea, Trunk)	Tsacahuico (Ch)	CH61
Hernandiaceae			
*Sparattanthelium amazonum* Mart.	MEDVET: Digestive system (Stomach ache, Trunk)	Nishi Tsanóna (Ch)	CH62
Hippocrateaceae			
*Cheiloclinum cognatum* (Miers.) A.C. Smith	MEDVET: Digestive system (Diarrhea, Bark); General Ailments with Unspecific Symptoms (Vomit, Bark and root); Musculo-skeletal system (Bone pain, Bark; Rheumatism, Bark); Respiratory system (Cold and flu, Bark)	Chuchuasa (Sp)	CH63
Hypericaceae			
*Vismia glaziovii* Ruhland	CONST: Houses (Muchacho - Ninotí, Trunk; Ridgepole - Maracatí, Trunk; Tirante - Cano bëpotó, Trunk)	Bisatamanë (Ch)	CH64
*Vismia macrophylla* Kunth	CONST: Houses (Chira Xahui, Trunk; Frame house, Bark; House post- Jibamë, Trunk; Jënë Jabati, Trunk; Jihuixaca, Trunk; Manipoatí, Trunk; Muchacho - Ninotí, Trunk; Nasëcamëti , Trunk; Pasa ratón - Xoya jabatí, Trunk; Ridgepole - Maracatí, Trunk; Roof beam - Canoxoco, Trunk; Solera - Chitao, Trunk; Tëtëmatsisi, Trunk; Tie - Xahui, Bark; Tirante - Cano bëpotó, Trunk; Tirante corto - Cano Bësëcamë, Trunk; Tirante largo - Cano pixquëna, Trunk; To tie house, Bark); Other constructions (Huaracha, Trunk); Thatch (To tie roof, Bark); CULT: Personal adornment (Ornament - Maxëití, Seeds; Ornament - Shinoxëta, Seeds); FUEL: Firewood (Firewood - Caro, Trunk); HUMFOOD: Food (Edible, Fruit); MEDVET: Digestive system (Malaria and fever, Leaf; Stomach ache, Leaf); Endocrine system (Liver pain, Leaf and seeds); Infections and infestations (Malaria and fever, Bark and leaf); Not specified at all (Cancer, Bark); Skin and subcutaneous tissue (Basket - Nishicacano, Trunk and exhudate; Haemorrhage, Bark); UTEN&TOOL: Domestic utensils (Basket - Chichabëcasa, Bark; Basket - Coquita, Bark; Basket - Nishicacano, Bark; Basket - Nishicacano, Trunk; Man basket, Bark; Tacú - Arusa timatí, Trunk); Labour tools (Hoe, Trunk)	Bisatamanë / Sipó / Sirari / Sisi (Ch); Palo Santo (Sp)	GS 48
*Vismia pozuzoensis* Engl.	CONST: Houses (Jihuixaca, Trunk; Muchacho - Ninotí, Trunk; Ridgepole - Maracatí, Trunk; Tirante - Cano bëpotó, Trunk; Tirante largo - Cano pixquëna, Trunk)	Bisatamanë / Jihui bapia (Ch); Leche leche / Piraquina (Sp)	DOA 17, 59, MSM 16, SCO 13
Lamiaceae			
*Clerodendrum tessmannii* Moldenke	FUEL: Firewood (Firewood - Caro, Trunk); HUMFOOD: Food (Edible, Fruit); MEDVET: Digestive system (Diarrhea, Bark, fruit and leaf; Stomach ache, Leaf); General Ailments with Unspecific Symptoms (Vomit, Bark, fruit and leaf); Respiratory system (Cold and flu, Leaf); Skin and subcutaneous tissue (Puchichi, Leaf); Urinary system (Kidney infection, Bark)	Guayagua (Ch); Guayaba (Sp)	BCM 9
*Vitex triflora* Vahl	FUEL: Firewood (Firewood - Caro, Trunk); MEDVET: Sensory system (Earache, Trunk)	Chaxo paoquí / Chaxo romë (Ch)	DOA 25, JSM 27
V*itex* sp.	MEDVET: Veterinary (Distemper, Bark)	Iene carama (Ch)	MOV 62
Lauraceae			
*Nectandra* sp.	FUEL: Firewood (Firewood - Caro, Trunk)	Xanë Yobini (Ch)	MOV 40
*Ocotea diospyrifolia* aff. (Meisn.) Mez	HUMFOOD: Food (Edible, Fruit); MEDVET: Skin and subcutaneous tissue (Caracha, Bark)	Nahuëshí (Ch)	CH65
*Persea americana* Mill.	HUMFOOD: Food (Edible, Fruit); MEDVET: General Ailments with Unspecific Symptoms (Vomit, Leaf); Infections and infestations (Leishmaniasis, Young leaf); Musculo-skeletal system (Hip pain, Leaf); Urinary system (Kidney pain, Leaf, root and seeds; Kidneys, Fruit, leaf, seeds and trunk)	Xane yubini cuota (Ch); Palta (Sp)	JSM 29
Lecythidaceae			
*Bertholletia excelsa* Bonpl.	CONST: Houses (Hedge - Panë, Trunk; Jihuixaca, Trunk; Muchacho - Ninotí, Trunk; Ridgepole - Maracatí, Trunk; Solera - Chitao, Trunk; Tirante - Cano bëpotó, Trunk; Tirante largo - Cano pixquëna, Trunk); Other constructions (Huaracha, Trunk); CULT: Dyes (Dye, Seeds); Personal adornment (Ornament - Maxëití, Seeds); HUMFOOD: Beberages (Beberage - Milk, Seeds); Food (Edible, Seeds); Oils (Oil, Seeds); MEDVET: Digestive system (Appendicitis, Seeds; Diarrhea, Seeds; Stomach ache, Seeds); Pregnancy, birth and puerperial (Haemorrhage after childbirth, Seeds); Skin and subcutaneous tissue (Caracha, Seeds; Haemorrhage, Bark, leaf and seeds; Wounds and cuts, Seeds); Urinary system (Gallstones, Seeds); UTEN&TOOL: Domestic utensils (Batán - Xaxo, Trunk)	Tapa / Tapa ristí / Tsixo (Ch); Almendro (Sp)	SCO 16
*Eschweilera albiflora* L.	CONST: Houses (To tie house, Bark); Thatch (Roof - Xëhuahacacató, Trunk; To tie roof, Bark); FUEL: Firewood (Firewood - Caro, Trunk); MEDVET: Musculo-skeletal system (Fractures, Bark); UTEN&TOOL: Domestic utensils (Basket - Chichama, Bark; Hammock - Nishi, Bark)	Maquë Tashi (Ch); Bitiumbo de bajio / Bitumbo / Campanilla / Cuchi / Piraquina (Sp)	MOV 58
*Eschweilera* sp.	HUMFOOD: Food (Edible, Fruit)	Tapa (Ch); Almendro (Sp)	MOV 51
*Gustavia hexapetala* (Aubl.) Sm.	CONST: Houses (Frame house, Trunk; Hedge - Panë, Trunk; House post- Jibamë, Trunk; Jihuixaca, Trunk; Manipoatí, Trunk; Muchacho - Ninotí, Trunk; Pasa ratón - Xoya jabatí, Trunk; Ridgepole - Maracatí, Trunk; Roof beam - Canoxoco, Trunk; Sabrillo, Trunk; Solera - Chitao, Trunk; Tirante - Cano bëpotó, Trunk; Tirante largo - Cano pixquëna, Trunk); Other constructions (Huaracha, Trunk); Thatch (Roof - Xëhuahacacató, Trunk); HUMFOOD: Food (Edible, Fruit); MEDVET: Sensory system (Inflammation of eyes, Root); UTEN&TOOL: Domestic utensils (Batán - Xaxo, Trunk; Pestle of Batan - Chapi, Trunk; Pestle of Tacu, Trunk; Spinning wheel - Ihui, Bark; Tacú - Arusa timatí, Trunk); Labour tools (Hammer, Trunk; Planting stick - Xësati, Trunk)	Yunishi (Ch); Itauba (Sp)	CH66
*Lecythis serrata* S.A. Mori	CULT: Ritual (Santeria, Seeds); MEDVET: Digestive system (Diarrhea, Seeds); Skin and subcutaneous tissue (Haemorrhage, Seeds)	Tapa (Ch); Almendro (Sp)	MSM 7
*Lecythis* sp.1	HUMFOOD: Beberages (Beberage - Milk, Fruit); Food (Edible, Fruit); Oils (Oil, Fruit); MEDVET: Skin and subcutaneous tissue (Haemorrhage, Fruit)	Almendro (Sp)	ESR 18
*Lecythis* sp.2	MEDVET: Not specified at all (Not specified, Fruit); Skin and subcutaneous tissue (Puchichi, Fruit)	Tapa (Ch)	GOS 34
Loganiaceae			
*Strychnos* sp.	HUMFOOD: Food (Edible, Fruit); MEDVET: General Ailments with Unspecific Symptoms (Headache, Leaf); Urinary system (Kidneys, Leaf)	Huani Kuhuësa (Ch)	GOS 28
Loranthaceae			
*Phthirusa pyrifolia* (Kunth) Eichler	FUEL: Firewood (Firewood - Caro, Trunk); HUMFOOD: Food (Edible, Fruit); MEDVET: Endocrine system (Liver, Bark and leaf); Musculo-skeletal system (Fractures, Bark and leaf)	Nishi moishi (Ch); Suelda con suelda (Sp)	DOA 41, 58, GOS 60, JSM 31
Lythraceae			
*Physocalymma scaberrimum* Pohl	UTEN&TOOL: Domestic utensils (Batán - Xaxo, Trunk)	Chaquillo (Sp)	JSM 46, RBU 17851
Malpighiaceae			
*Bunchosia glandulifera* (Jacq.) Kunth	HUMFOOD: Food (Edible, Fruit)	Mermelada (Sp)	ESR 21
*Byrsonima crispa* A. Juss.	FUEL: Firewood (Firewood - Caro, Trunk)	Xëchi (Ch)	CH67
*Heteropterys coriacea* A. Juss.	CONST: Houses (Hedge - Panë, Trunk; House post- Jibamë, Trunk); Other constructions (Huaracha, Trunk)	Xaba yunishi (Ch)	JSM 2, RBU 17808
*Mascagnia macrophylla* Rusby	UTEN&TOOL: Domestic utensils (Bow to clean cotton, Trunk)	Ascana (Ch)	CH68
Malvaceae			
*Apeiba tibourbou* Aubl.	CONST: Houses (Frame house, Bark; Hedge - Panë, Trunk; Muchacho - Ninotí, Trunk; Pasa ratón - Xoya jabatí, Trunk; Ridgepole - Maracatí, Trunk; Roof beam - Canoxoco, Trunk; Tie - Xahui, Bark; Tirante - Cano bëpotó, Trunk; Tirante largo - Cano pixquëna, Trunk; To tie fence, Bark); Other constructions (Huaracha, Trunk); Thatch (To tie roof, Bark); CULT: Clothes & accessories (Dress - Moro, Bark); FUEL: Firewood (Firewood - Caro, Trunk); Other fuel (Ceramics - Chomo, Bark); HUMFOOD: Food (Edible, Fruit); MEDVET: General Ailments with Unspecific Symptoms (Headache, Bark); Sensory system (Earache, Young leaf); Skin and subcutaneous tissue (Caracha, Bark); UTEN&TOOL: Domestic utensils (Basket - Bano, Bark; Basket - Cacachuquëxnia, Bark; Basket - Chichama, Bark; Basket - Nishicacano, Bark; Basket - Purupachi, Bark; Basket - Yamachi, Bark; Hammock - Nishi, Bark); Hunting & fishing tools (Bow - Canatí, Trunk); Rope (Rope - Rispichi, Bark)	Bitumbo (Sp)	DOA 44, GOS 25, JSM 60, RBU 17867
*Eriotheca* sp.	MEDVET: Infections and infestations (Malaria and fever, Bark); UTEN&TOOL: Domestic utensils (Pestle of Tacu, Trunk; Tacú - Arusa timatí, Trunk)	Iso nareja (Ch)	DOA 18
*Gossypium barbadense* L.	CONST: Houses (Tie - Xahui, Bark); CULT: Clothes & accessories (Dress - Moro, Seeds); Personal adornment (Ornament - Amënoxëta, Seeds; Ornament - Baxëxëtí, Seeds; Ornament - Chaha, Seeds; Ornament - Chinoxëta , Seeds; Ornament - Chua, Seeds; Ornament - Matsamití, Seeds; Ornament - Maxëití, Seeds; Ornament - Mënëxëtí, Seeds; Ornament - Rësëti, Seeds; Ornament - Shinoxëta, Seeds; Ornament - Tsirispi, Seeds; Ornament - Xapo, Seeds); Recreational (Toys, Seeds); FUEL: Firewood (Firewood - Caro, Trunk); HUMFOOD: Food (Edible, Seeds); MEDVET: Digestive system (Diarrhea, Trunk); General Ailments with Unspecific Symptoms (Body pain, Trunk; Headache, Leaf and leaf; Vomit, Leaf); Musculo-skeletal system (Bone pain, Leaf); Respiratory system (Cold and flu, Leaf); Sensory system (Earache, Flower, leaf and seeds); Skin and subcutaneous tissue (Caracha, Leaf; Haemorrhage, Leaf; Puchichi, Leaf); Urinary system (Kidney pain, Leaf); UTEN&TOOL: Domestic utensils (Basket - Nishicacano, Bark; Hammock - Nishi, Seeds); Hunting & fishing tools (Arrow, Seeds; Fishing lines, Seeds); Labour tools (Planting stick - Xësati, Trunk); Rope (Rope - Rispichi, Seeds; Rope, Seeds)	Algodón (Sp)	GCM 8, GOS 11, MOV 21, ORC 6
*Lueheopsis schultesii* Cuatrec.	CULT: Personal adornment (Ornament - Huaxmënëhua, Seeds)	Huaxmënëhua (Ch)	CH69
*Ochroma pyramidale* (Cav. ex Lam.) Urb.	CONST: Houses (Hedge - Panë, Trunk; Tie - Xahui, Bark; To tie house, Bark); Other constructions (Huaracha, Trunk); Thatch (To tie roof, Bark); CULT: Recreational (Toys, Trunk; Zampoña - Bistó, Bark); HUMFOOD: Food (Edible, Fruit); MEDVET: General Ailments with Unspecific Symptoms (Chest pain, Seeds); Infections and infestations (Scabies); Skin and subcutaneous tissue (Caracha, Bark; Skin fungus, Bark); UTEN&TOOL: Domestic utensils (Basket - Cacachuquëxnia, Bark; Basket - Chichama, Bark; Basket - Nishicacano, Bark; Hammock - Nishi, Bark; Table, Trunk); Rope (Rope - Rispichi, Bark)	Balsa (Sp)	ESR 23, JSM 17
*Pseudobombax septenatum* (Jacq.) Dugand	FUEL: Firewood (Firewood - Caro, Trunk); MEDVET: General Ailments with Unspecific Symptoms (Headache, Leaf); Infections and infestations (Malaria and fever, Leaf)		DOA 36, JSM 57
*Theobroma grandiflorum* (Willd. ex Spreng.) K. Schum.	HUMFOOD: Food (Edible, Fruit)	Copoazú (Sp)	CH70
*Theobroma speciosum* (Willd. ex Spreng.) K. Schum.	ANIMFOOD: Fodder (Edible, Fruit); FUEL: Firewood (Firewood - Caro, Trunk); HUMFOOD: Food (Edible, Seeds); MEDVET: Infections and infestations (Malaria and fever, Leaf); Not specified at all (Not specified, Fruit); Sensory system (Earache, Flower)	Chocolate / Chocolatillo (Sp)	ESR 10, DOA 16, 39, GOS 21, JSM 5, 30, SCO 30
Marantaceae			
*Calathea* sp.	FUEL: Firewood (Firewood - Caro, Trunk); UTEN&TOOL: Wrappers (Wrappers, Leaf)	Manicoro (Ch); Japaina (Sp)	MOV 41
Melastomataceae			
*Bellucia acutata* Pilg.	HUMFOOD: Food (Edible, Fruit)	Guayabilla (Sp)	BCM 5, ESR 15, MOV 6
*Miconia albicans* (Sw.) Triana	FUEL: Firewood (Firewood - Caro, Trunk); MEDVET: Infections and infestations (Malaria and fever); Respiratory system (Cold and flu)	Blanquillo (Ch)	JSM 3, MOV 17, RBU 17818
*Miconia argyrophylla* DC.	HUMFOOD: Food (Edible, Fruit)		MSM 2
*Miconia nervosa* (Sm.) Triana	ANIMFOOD: Fodder (Edible, Fruit); CONST: Houses (Tirante largo - Cano pixquëna, Trunk)		DOA 26
*Miconia tiliifolia* Naudin	HUMFOOD: Food (Edible, Fruit)	Nigua (Ch)	ESR 2, RBU 17819, 17838
*Miconia* sp.	MEDVET: Skin and subcutaneous tissue (Puchichi, Fruit)	Pao (Ch)	DOS 60
*Mouriri guianensis* Aubl.	CULT: Ritual (Good luck, Leaf and whole plant); HUMFOOD: Food (Edible, Fruit); MEDVET: Respiratory system (Cold and flu, Flower); Skin and subcutaneous tissue (Puchichi); UTEN&TOOL: Domestic utensils (Pestle of Tacu, Trunk); Hunting & fishing tools (Arrow - Bicobi, Trunk; Arrow, Trunk; Bow - Canatí, Trunk); Labour tools (Planting stick - Xësati, Trunk)	Llave (Sp)	DOA 37
Meliaceae			
*Cedrela fissilis* Vell.	CONST: Other constructions (Huaracha, Trunk); CULT: Personal adornment (Ornament - Mënëxëtí, Bark); FUEL: Firewood (Firewood - Caro, Trunk); HUMFOOD: Food (Edible, Fruit); MEDVET: Cultural diseases and disorders (Bad air and scare - Ratëaina, Leaf); Digestive system (Diarrhea, Bark; Stomach ache, Bark); Endocrine system (Liver pain, Bark); General Ailments with Unspecific Symptoms (Vomit, Bark); Infections and infestations (Malaria and fever, Bark); UTEN&TOOL: Domestic utensils (Chair - Taburete, Trunk; Pestle of Tacu, Trunk; Spoon, Trunk; Table, Trunk); Other utensils (Boxes, Trunk)	Cedro (Sp)	CH71
Menispermaceae			
*Abuta grandifolia* (Mart.) Sandwich	HUMFOOD: Food (Edible, Fruit)		ESR 7
Moraceae			
*Brosimum gaudichaudii* Trécul	FUEL: Firewood (Firewood - Caro, Trunk); HUMFOOD: Food (Edible, Fruit); MEDVET: Not specified at all (Not specified, Bark); UTEN&TOOL: Domestic utensils (Tacú - Arusa timatí, Trunk)	Apta (Ch)	DOA 4, MOV 2
*Brosimum guianense* (Aubl.) Huber	FUEL: Firewood (Firewood - Caro, Trunk); HUMFOOD: Food (Edible, Fruit)		CH72
*Brosimum utile* subsp. *ovatifolium* (Ducke) C.C. Berg	CONST: Other constructions (Huaracha, Bark); CULT: Clothes & accessories (Dress - Pío, Bark); UTEN&TOOL: Domestic utensils (Raití, Bark); Tacú - Arusa timatí, Trunk (Hunting & fishing tools)	Pío (Ch); Bibosi (Sp)	CH73
*Chlorophora tinctoria* (L.) Gaudich. ex Benth.	ANIMFOOD: Fodder (Edible, Fruit); CONST: Houses (Tirante largo - Cano pixquëna, Trunk)	Nibosa (Ch)	CH74
*Ficus gomelleira* Kunth & C.D. Bouché	CULT: Clothes & accessories (Dress - Isaca pohi, Bark; Dress - Moro, Bark); Recreational (Comëno, Bark); MEDVET: Musculo-skeletal system (Fractures, Bark; Fractures, Exudate); Skin and subcutaneous tissue (Wounds and cuts, Bark); UTEN&TOOL: Domestic utensils (Pestle of Tacu, Trunk; Raití, Bark); Rope (Rope - Rispichi, Bark)	Matapalo (Sp)	CH75
*Ficus gomelleria* Kunth & C.D. Bouché	CULT: Clothes & accessories (Dress - Xóa, Bark)	Xoá (Ch); Bibosi blanco (Sp)	CH76
*Ficus mathewsii* (Miq.) Miq.	CULT: Clothes & accessories (Dress - Isaca pohi, Bark); MEDVET: Insect and athropod bites (Buna bite, Bark); CONST: Houses (To tie house, Bark); Other constructions (Huaracha, Trunk); CULT: Clothes & accessories (Dress - Moro, Bark; Dress - Mororia, Bark); Personal adornment (Ornament - Amënoxëta, Seeds; Ornament - Rësëti; Ornament - Shinoxëta, Bark; Ornament - Xapo, Bark); FUEL: Firewood (Firewood - Caro, Trunk); MEDVET: Dental health (Toothache, Exudate); Infections and infestations (Malaria and fever, Bark); Musculo-skeletal system (Fractures, Bark and exhudate); Skin and subcutaneous tissue (Wounds and cuts, Trunk); UTEN&TOOL: Domestic utensils (Basket - Yamachi, Bark; Batán - Xaxo, Trunk; Chair - Taburete, Trunk; Hammock - Nishi, Bark); Hunting & fishing tools (Bow - Canatí, Bark); Rope (Rope - Rispichi, Bark; Rope, Bark)		GOS 50, MOA 7
*Ficus sphenophylla* Standl.	CULT: Clothes & accessories (Dress - Isaca pohi, Bark); MEDVET: Insect and athropod bites (Buna bite, Exudate); CONST: Other constructions (Huaracha, Bark); CULT: Clothes & accessories (Dress - Moro, Bark); Personal adornment (Ornament - Xapo, Bark); MEDVET: Musculo-skeletal system (Fractures, Exudate)	Isaca Pohi (Ch); Bibosi (Sp)	CH77
*Ficus trigona* L.f.	CONST: Other constructions (Huaracha, Bark); CULT: Clothes & accessories (Dress - Moro, Bark); Personal adornment (Ornament - Xapo, Bark); MEDVET: Musculo-skeletal system (Fractures, Exudate)	Moro (Ch); Bibosi (Sp)	JSM 51
*Ficus* sp.	CONST: Houses (To tie house, Bark); Other constructions (Huaracha, Trunk); CULT: Clothes & accessories (Dress - Moro, Bark; Dress - Mororia, Bark); Personal adornment (Ornament - Amënoxëta, Seeds; Ornament - Rësëti; Ornament - Shinoxëta, Bark; Ornament - Xapo, Bark); FUEL: Firewood (Firewood - Caro, Trunk); MEDVET: Dental health (Toothache, Exudate); Infections and infestations (Malaria and fever, Bark); Musculo-skeletal system (Fractures, Bark and exhudate); Skin and subcutaneous tissue (Wounds and cuts, Trunk); UTEN&TOOL: Domestic utensils (Basket - Yamachi, Bark; Batán - Xaxo, Trunk; Chair - Taburete, Trunk; Hammock - Nishi, Bark); Hunting & fishing tools (Bow - Canatí, Bark); Rope (Rope - Rispichi, Bark; Rope, Bark)	Mororía (Ch); Bibosi (Sp)	CH78
*Helicostylis tomentosa* (Poepp. & Endl.) Rusby	FUEL: Firewood (Firewood - Caro, Trunk); HUMFOOD: Food (Edible, Fruit); MEDVET: Insect and athropod bites (Buna bite, Exudate; Insectbite, Exudate); Skin and subcutaneous tissue (Wounds and cuts, Exudate)	Nui (Sp)	DOA 15, MOV 42, SCO 26
*Perebea angustifolia* (Poepp. & Endl.) C. C. Berg	FUEL: Firewood (Firewood - Caro, Trunk); HUMFOOD: Food (Edible, Fruit)		CH79
*Perebea mollis* (Poepp. & Endl.) Huber	FUEL: Firewood (Firewood - Caro, Trunk); HUMFOOD: Food (Edible, Fruit); MEDVET: Insect and athropod bites (Buna bite, Bark)	Patai Perro (Ch); Patamichi (Sp)	SCO 27, DOA 33
*Pseudolmedia macrophylla* Trécul	CONST: Houses (To tie house, Bark); FUEL: Firewood (Firewood - Caro, Trunk); HUMFOOD: Food (Edible, Fruit)	Maca Nui (Ch); Nui (Sp)	
*Pseudolmedia* sp.	FUEL: Firewood (Firewood - Caro, Trunk); HUMFOOD: Food (Edible, Fruit)	Roble de Pajo (Sp)	MOV 63, SCO 40
*Sorocea guilleminiana* Gaudich	CONST: Houses (Tie - Xahui, Bark; To tie house, Bark); HUMFOOD: Food (Edible, Fruit)		
*Sorocea muriculata* Miq.	CULT: Personal adornment (Ornament - Maxëití, Fruit); Ritual (Marriage ceremony; Symbology, Trunk)		DOA 56, GOS 55
Musaceae			
*Musa x paradisiaca* L.	HUMFOOD: Beberages (Beberage - Chicha, Fruit; Beberage , Fruit); Food (Edible - Chipilo, Fruit; Edible - Chive, Fruit; Edible, Fruit); MEDVET: Dental health (Blisters mouth, Fruit; Toothache, Root); Digestive system (Diarrhea, Exudate); Skin and subcutaneous tissue (Burns, Fruit; Empeine, Fruit); UTEN&TOOL: Domestic utensils (Basket - Bano, Leaf)	Bochi / Carapë / Carapë Mëxti / Carapëi / Carapëna / Carapëria / Jënë Jabati / Macho / Mëxti / Naraja Carapë / Pia / Sano / Shica Carapë / Tëtëca / Tëtoyá / Tumichuqua (Ch); Banana / Guineo / Guineo Beromo / Guineo isleño / Guineo mataborracho / Guineo morado / Guineo motacusillo / Guineo seda / Plátano / Plátano Bellaco / Plátano chama / Plátano largo / Plátano Motacusillo / Seda / Seda berda / Seda morado (Sp)	
Myristicaceae			
*Iryanthera juruensis* Warb.	FUEL: Firewood (Firewood - Caro, Trunk); HUMFOOD: Food (Edible, Fruit); MEDVET: Dental health (Thrush, Exudate); Skin and subcutaneous tissue (Caracha, Exudate)	Bita / Sangre de Toro (Sp)	CH80
*Iryanthera* sp.	CONST: Houses (Tie - Xahui, Bark); FUEL: Firewood (Firewood - Caro, Trunk); HUMFOOD: Food (Edible, Fruit); MEDVET: Insect and athropod bites (Insectbite)	Bach rao (Ch)	CH81
*Virola flexuosa* A.C. Sm.	MEDVET: Musculo-skeletal system (Fractures, Bark and leaf)	Suelda con suelda (Sp)	JSM 32
*Virola sebifera* Aubl.	CULT: Cosmetic (Hair oil, Exudate)	Toro (Ch)	CH82
Myrtaceae			
*Eugenia* sp. 1	HUMFOOD: Food (Edible, Fruit)		RBU 17824
*Eugenia* sp. 2	MEDVET: Skin and subcutaneous tissue (Caracha)	Mëtëquë (Ch)	JSM 12
*Myrcia mollis* (Kunth) DC.	FUEL: Firewood (Firewood - Caro, Trunk)		BCM 8
*Myrcia regnelliana* O. Berg	MEDVET: Skin and subcutaneous tissue (Caracha, Bark and fruit; Wounds and cuts, Bark and root)		CH83
*Myrciaria floribunda* (H. West ex Willd.) O. Berg	MEDVET: General Ailments with Unspecific Symptoms (Chest pain, Bark); Musculo-skeletal system (Cramps, Bark, Rheumatism, Bark)	Hoja del monte (Ch)	CH84
*Psidium guajava* L.	MEDVET: Infections and infestations (Malaria and fever, Bark)	Bahua quëxti (Ch)	BCM 10, ORC 1
Ochnaceae			
*Ouratea angulata* Tiegh.	MEDVET: Musculo-skeletal system (Rheumatism, Bark)	Chuchuhuaso monte alto (Sp)	ESR 8
*Ouratea* sp.	FUEL: Firewood (Firewood - Caro, Trunk)	Xaba pëpëcho (Ch)	JSM 10
Olacaceae			
*Minquartia guianensis* Aubl.	CONST: Houses (House post- Jibamë, Trunk); MEDVET: Skin and subcutaneous tissue (Hand blisters, Bark)	Cacharí (Ch)	GOS 9
Oxalidaceae			
*Agonandra b rasiliensis* Miers ex Benth.	HUMFOOD: Food (Edible, Fruit)	Aceituna (Sp)	CH85
*Averrhoa carambola* L.	HUMFOOD: Food (Edible, Fruit)	Carambola (Sp)	
Passifloraceae			
*Passiflora coccinea* Aubl.	UTEN&TOOL: Hunting & fishing tools (Barbasco - Iscoró, Trunk)		CH86
*Passiflora miniata* Aubl.	HUMFOOD: Food (Edible, Fruit)	Pachio fuerte (Sp)	MOV 10, SCO 21
*Passiflora tripartita* (Juss.) Poir.	UTEN&TOOL: Domestic utensils (Basket - Nishicacano, Trunk)	Tumbo (Sp)	CH87
Piperaceae			
*Peperomia pellucida* (L.) Kunth	MEDVET: General Ailments with Unspecific Symptoms (Chest pain, Fruit)		CH88
*Piper bartlingianum* (Miq.) C. DC.	MEDVET: Skin and subcutaneous tissue (Caracha, Root); UTEN&TOOL: Domestic utensils (Basket - Bano)		JSM 34, MSM 8
*Piper hispidum* Sw.	MEDVET: Dental health (Toothache, Root); Not specified at all (Operations, Trunk); Skin and subcutaneous tissue (Burns, Leaf; Caracha, Leaf, root, trunk and whole plant; Wounds and cuts, Leaf and trunk)		SCO 10, GOS 12
*Piper nigrispicum* Sw.	MEDVET: Digestive system (Stomach ache, Bark); Endocrine system (Liver pain, Trunk); Sensory system (Inflammation of eyes)	Nishipara / Yunquilla (Ch)	CH89
*Piper peltatum* Sw.	MEDVET: General Ailments with Unspecific Symptoms (Vomit, Leaf); Respiratory system (Cold and flu, Bark and leaf); Sensory system (Earache, Root)	Boca de Hombre (Sp)	GOS 43, MOV 60
*Piper piscatorum* Sw.	MEDVET: Dental health (Toothache, Exudate, root and trunk); Digestive system (Diarrhea, Trunk); Musculo-skeletal system (Bone pain, Root); Not specified at all (Not specified, Root); Skin and subcutaneous tissue (Caracha, Leaf)	Nucaperi (Ch)	DOA 7
P*iper* sp.	MEDVET: Respiratory system (Cold and flu, Whole plant); Urinary system (Kidney pain, Whole plant)	Matico (Sp)	MOA 9
Poaceae			
*Cymbopogon citratus* (DC.) Stapf	MEDVET: Digestive system (Stomach ache, Leaf); General Ailments with Unspecific Symptoms (Vomit, Leaf); Infections and infestations (Malaria and fever, Leaf and root); Pregnancy, birth and puerperial (Accelerator for birth, Root; Birth, Leaf; Haemorrhage after childbirth, Leaf); Reproductive system and sex health (Menstrual pain, Leaf); Respiratory system (Cold and flu, Leaf); Sensory system (Inflammation of eyes, Leaf); Skin and subcutaneous tissue (Haemorrhage, Leaf)	Cedrón (Sp)	
*Guadua* sp. 1	UTEN&TOOL: Domestic utensils (Chair - Taburete, Trunk)		DOA 22
*Guadua* sp. 2	CULT: Recreational (Zampoña - Bistó, Trunk); MEDVET: Pregnancy, birth and puerperial (To cut umbilical cord, Trunk); UTEN&TOOL: Domestic utensils (Knife, Trunk)		MOV 57
*Gynerium sagittatum* (Aubl.) P. Beauv.	CONST: Houses (Hedge - Panë, Trunk); CULT: Clothes & accessories (Comb, Trunk); Personal adornment (Ornament - Maxëití, Seeds; Ornament - Rësëti, Trunk); Recreational (Zampoña - Bistó, Trunk); MEDVET: Musculo-skeletal system (Fractures); Snakebites and Ray stings (Sankebites, Trunk); UTEN&TOOL: Domestic utensils (Mat, Leaf); Hunting & fishing tools (Arrow - Bicobi, Trunk; Arrow - Notsi, Trunk; Arrow - Pio, Trunk; Arrow - Quërëquë, Trunk; Arrow - Quëspini, Trunk; Arrow - Tahua Quëspini, Trunk; Arrow - Tiopi, Trunk; Arrow, Fruit; Bow - Canatí, Trunk; Weapons, Trunk)	Tacuara (Ch); Chuchío / Paja corona (Sp)	CH90
*Gynerium* sp.	UTEN&TOOL: Domestic utensils (Knife, Trunk)		MOV 30
*Lasiacis ligulata* Hitchc. & Chase.	CULT: Recreational (Zampoña - Bistó, Trunk); MEDVET: Dental health (Blisters mouth, Fruit)	Tacuarilla (Ch)	CH91
*Olyra micrantha* Kunth	CULT: Personal adornment (Ornament - Matsamití, Trunk; Ornament - Rësëti, Trunk); Recreational (Zampoña - Bistó, Trunk); FUEL: Other fuel (Ceramics - Comëno, Trunk); UTEN&TOOL: Hunting & fishing tools (Arrow - Bicobi, Trunk; Arrow - Notsi, Trunk; Arrow - Quërëquë, Trunk; Arrow - Quëspini, Trunk; Arrow - Tahua Quëspini, Trunk; Arrow - Tiopi, Trunk)	Tacuarilla (Ch)	CH92
*Oryza sativa* L.	CULT: Recreational (Zampoña - Bistó, Trunk); HUMFOOD: Food (Edible, Seeds)	Arroz (Sp)	
*Pharus latifolius* L.	UTEN&TOOL: Hunting & fishing tools (Barbasco - Axa, Trunk)	Jënë arosa (Ch); Barbasco (Sp)	CH92
*Saccharum officinarum* L.	CONST: Houses (House post- Jibamë, Trunk); FUEL: Firewood (Firewood - Caro, Trunk); HUMFOOD: Beberages (Beberage , Trunk); Food (Edible, Trunk); MEDVET: Urinary system (Kidneys, Trunk)	Shita chëque / Shita sihoyá / Shitaria / Shitatë (Ch); Caña (Sp)	
*Streptogyna americana* C.E. Hubb	CONST: Houses (Frame house, Trunk); MEDVET: Endocrine system (Liver pain, Root); General Ailments with Unspecific Symptoms (Vomit, Root); Urinary system (Kidney infection, Root; Kidneys, Root); Veterinary (Distemper, Root)	Huasimapoa (Ch); Sujo (Sp)	CH93
*Zea mays* L.	ANIMFOOD: Fodder (Edible, Seeds); HUMFOOD: Beberages (Beberage - Chicha, Seeds; Beberage - Wiñapo, Seeds); Food (Edible - Chive, Seeds; Edible - Flour, Seeds; Edible - Tamales, Seeds; Edible, Seeds)	Cahuayo Xëqui / Canashibati / Chítoco / Itëma / Jimi Xëqui / Xëqui / Xëqui bëtëmë / xëqui joxo / Xëquiria / Xino xëqui (Ch); Maíz / máiz amarillo / Maíz blanco / Maíz colorado / Maíz corto / Maíz cubano / Maíz negro (Sp)	
Polygalaceae			
*Bredemeyera myrtifolia* Spruce ex A.W. Benn.	CULT: Personal adornment (Ornament - Shinoxëta, Trunk)	Bahuino Nihi (Ch)	BCM 4, ESR 3
Polygonaceae			
*Triplaris americana* L.	MEDVET: Digestive system (Diarrhea, Bark and leaf; Stomach ache, Seeds); General Ailments with Unspecific Symptoms (Chest pain; Headache, Seeds; Vomit, Leaf); Infections and infestations (Malaria and fever, Bark and leaf); Musculo-skeletal system (Fractures, Bark); Respiratory system (Cough)	Janina (Ch); Palo Diablo (Sp)	GOS 47, JSM 58, MOA 5, MOV 59, ORC 2
Polypodiaceae			
*Phlebodium decumanum* (Willd.) J. Sm.	MEDVET: Infections and infestations (Leishmaniasis, Bark); Respiratory system (Cold and flu, Bark); Skin and subcutaneous tissue (Caracha, Root); Urinary system (Kidneys, Bark, leaf and root)	Roho jina (Ch); Cola de manechi (Sp)	DOA 9, ORC 4
Proteaceae			
*Roupala* sp.	MEDVET: Sensory system (Earache, Leaf)	Mahi No Nihi (Ch)	CH94
Pteridaceae			
*Adiantum latifolium* Lam.	CONST: Houses (Tie - Xahui, Bark; To tie house, Trunk); Thatch (Roof - Xëhuahacacató, Trunk; To tie roof, Trunk); CULT: Personal adornment (Ornament - Mënëxëtí, Trunk; Ornament - Shinoxëta); UTEN&TOOL: Domestic utensils (Basket - Nishicacano, Trunk)	Mitaisa (Ch); Bejuco (Sp)	MOV 67
*Adiantum lucidum* (Cav.) Sw.	CONST: Houses (To tie house, Trunk); MEDVET: Skin and subcutaneous tissue (Caracha, Exudate)	Mitsisi (Ch)	CH95
*Adiantum obliquum* Willd.	MEDVET: Not specified at all (Not specified); UTEN&TOOL: Rope (Rope - Rispichi, Trunk)	Mitsisi (Ch)	CH96
*Adiantum petiolatum* Desv.	MEDVET: Infections and infestations (Scabies); Skin and subcutaneous tissue (Skin infection)	Mitsisi (Ch)	CH97
*Pteris* sp.	MEDVET: Urinary system (Kidney infection, Trunk)	Bushishi (Ch); Shico (Sp)	DOA 50
Rosaceae			
*Prunus amplifolia* Pilger	CONST: Houses (Hedge - Panë, Trunk; Roof beam - Canoxoco, Trunk); FUEL: Firewood (Firewood - Caro, Trunk); UTEN&TOOL: Hunting & fishing tools (Bow - Canatí, Trunk); Labour tools (Planting stick - Xësati, Trunk; Shovel, Trunk)	Jihui (Ch)	CH98
Rubiaceae			
*Alibertia edulis* (Rich.) A. Rich. ex DC.	HUMFOOD: Food (Edible, Fruit); MEDVET: Digestive system (Diarrhea, Fruit and leaf); General Ailments with Unspecific Symptoms (Vomit)	Tosa (Ch); Guayabilla / Tutumilla (Sp)	ESR 12, MOV 1, 15
*Amaioua guianensis* Aubl.	FUEL: Firewood (Firewood - Caro, Trunk); UTEN&TOOL: Labour tools (Axe - Maquë poroma, Trunk; Machete handle, Trunk; Planting stick - Xësati, Trunk)	Cai Osho (Ch)	DOA 32
*Capirona decorticans* Spruce	FUEL: Firewood (Firewood - Caro, Trunk); HUMFOOD: Food (Edible, Fruit); MEDVET: Infections and infestations (Scabies, Bark); Insect and athropod bites (Buna bite, Bark; Insectbite, Bark); Not specified at all (Cepta, Bark); Reproductive system and sex health (Contraceptive, Bark); Respiratory system (Cold and flu, Bark and flower); Skin and subcutaneous tissue (Burns, Bark; Caracha, Bark; Haemorrhage, Bark; Wounds and cuts, Bark); Snakebites and Ray stings (Sankebites, Bark); UTEN&TOOL: Domestic utensils (Pestle of Tacu, Trunk); Labour tools (Planting stick - Xësati, Trunk; Shovel, Trunk)	Batahua (Ch); Guayabochi (Sp)	DOA 28, ESR 14, JSM 22, MOV 36, RBU 17823, SCO 39
*Coutarea hexandra* (Jacq.) K. Schum.	MEDVET: Digestive system (Diarrhea, Bark, leaf and root; Stomach ache, Bark); Endocrine system (Gallbladder, Bark); Infections and infestations (Malaria and fever, Bark); Reproductive system and sex health (Abortive, Leaf)	Jihui Moca (Ch)	CH99
*Genipa americana* L.	CULT: Personal adornment (Ornament - Maxëití, Seeds); HUMFOOD: Food (Edible, Fruit)	Nanë (Ch); Bii (Sp)	JSM 6
*Geophila cordifolia* Miq.	MEDVET: Infections and infestations (Amoebas, Leaf; Anthelmintic, Leaf)	Mai yochi (Ch)	DOA 35
*Ladenbergia oblongifolia* (Mutis) L. Anderes	HUMFOOD: Food (Edible, Fruit)	Muela (Sp)	CH100
*Palicourea rigida* Kunth	HUMFOOD: Food (Edible, Fruit); MEDVET: Skin and subcutaneous tissue (Caracha, Trunk)	Áhuara Macha (Ch)	MOV 13
*Psychotria deflexa* DC.	CONST: Houses (To tie house, Bark)	Yotabi (Ch)	CH101
*Psychotria iodotricha* Müll. Arg.	HUMFOOD: Food (Edible, Fruit)	Bimi Chëxë (Ch)	CH102
*Psychotria lupulina* Benth.	MEDVET: Infections and infestations (Boro, Bark)	Ahuara Nihi (Ch)	CH103
*Psychotria prunifolia* (Kunth) Steyerm.	MEDVET: Digestive system (Diarrhea, Bark); General Ailments with Unspecific Symptoms (Vomit, Bark)	Bimi Chëxë (Ch)	CH104
*Psychotria* sp. 1	CULT: Personal adornment (Ornament - Maxëití, Fruit)	Bimi Chëxë (Ch)	JSM 25
*Psychotria* sp. 2	CULT: Personal adornment (Ornament - Maxëití, Fruit); MEDVET: Digestive system (Diarrhea, Root)	Bimi Chëxë (Ch)	DOA 14
*Randia armata* cf. (Sw.) DC.	FUEL: Firewood (Firewood - Caro, Trunk)	Pata de Gallina (Sp)	CH105
*Uncaria guianensis* (Aubl.) J.F. Gmel.	CULT: Personal adornment (Ornament - Matsamití, Seeds); FUEL: Other fuel (Ceramics - Chomo, Trunk); MEDVET: Digestive system (Diarrhea, Bark; Stomach ache, Bark); Endocrine system (Liver pain, Bark); General Ailments with Unspecific Symptoms (Chest pain, Bark; Headache, Bark; Inflammation, Bark; Vomit, Bark); Musculo-skeletal system (Bone pain, Bark; Fractures, Bark; Hip pain, Bark); Respiratory system (Cold and flu, Bark); Urinary system (Kidney infection, Bark); UTEN&TOOL: Domestic utensils (Basket - Cacachuquëxnia, Trunk; Basket - Chichama, Bark; Basket - Nishicacano, Trunk; Basket - Purupachi, Bark; Basket - Yamachi, Trunk)	Cacatho (Ch); Uña de Gato (Sp)	ESR 6, 20, JSM 53, MOV 3, SCO 7
Indet. sp. 1	CONST: Houses (To tie house, Bark); Thatch (To tie roof, Bark)		
Rutaceae			
*Citrus aurantiifolia* (Christm.) Swingle	FUEL: Firewood (Firewood - Caro, Trunk); HUMFOOD: Food (Edible, Fruit); MEDVET: Dental health (Toothache, Seeds); Digestive system (Diarrhea, Root; Stomach ache, Fruit and root); Endocrine system (Liver pain, Fruit); General Ailments with Unspecific Symptoms (Chest pain, Fruit; Headache, Fruit; Vomit, Root); Infections and infestations (Malaria and fever, Bark); Musculo-skeletal system (Bone pain, Bark, leaf and root); Respiratory system (Cold and flu, Fruit and leaf); Sensory system (Inflammation of eyes, Seeds); Urinary system (Kidneys, Fruit)	Rimó (Ch); Limón (Sp)	
*Citrus limetta* Risso	MEDVET: Infections and infestations (Malaria and fever, Root)	Lima (Sp)	
*Citrus paradisi* Macfad.	CONST: Houses (To tie house, Bark); HUMFOOD: Food (Edible, Fruit); MEDVET: Digestive system (Diarrhea, Fruit; Stomach ache, Bark and leaf); General Ailments with Unspecific Symptoms (Vomit, Root); Infections and infestations (Malaria and fever, Bark); Skin and subcutaneous tissue (Caracha, Root; Wounds and cuts, Root); UTEN&TOOL: Domestic utensils (Basket - Chichama, Bark)	Taraja (Ch); Toronja (Sp)	
*Citrus reticulata* Blanco	HUMFOOD: Food (Edible, Fruit); MEDVET: Digestive system (Stomach ache, Leaf)	Mandarina (Sp)	
*Citrus* x *sinensis* (L.) Osbeck	HUMFOOD: Food (Edible, Fruit); MEDVET: Digestive system (Diarrhea, Bark, fruit and leaf); Endocrine system (Liver pain, Leaf); Reproductive system and sex health (Abortive, Root); Urinary system (Kidneys, Fruit); UTEN&TOOL: Domestic utensils (Pestle of Batan - Chapi, Trunk; Pestle of Tacu, Trunk)	Naraja (Ch); Naranja (Sp)	
*Metrodorea flavida* K. Krause	CONST: Houses (House post- Jibamë, Trunk; Muchacho - Ninotí, Trunk; Pasa ratón - Xoya jabatí, Trunk; Roof beam - Canoxoco, Trunk; Tie - Xahui, Bark; Tirante - Cano bëpotó, Trunk; Tirante largo - Cano pixquëna, Trunk); FUEL: Firewood (Firewood - Caro, Trunk); HUMFOOD: Food (Edible, Fruit); UTEN&TOOL: Domestic utensils (Pestle of Tacu, Trunk); Labour tools (Hammer, Trunk)	Paxa ini (Ch); Blanquillo / Palo Blanco / Palo Coca / Palo Coloradillo (Sp)	SCO 25
*Moniera trifolia* L.	CULT: Ritual (Fragile children, Leaf); MEDVET: Cultural diseases and disorders (Bad air and scare - Ratëaina, Leaf); Sensory system (Earache, Leaf)	Ororotso (Ch)	MSM 19
*Zanthoxylum rhoifolium* Lam.	FUEL: Firewood (Firewood - Caro, Trunk)	Coroquisi (Ch)	JSM 45
Salicaceae			
*Casearia arborea* (Rich.) Urb.	FUEL: Firewood (Firewood - Caro, Trunk); HUMFOOD: Beberages (Beberage - Chicha, Seeds)	Jihui pohi (Ch)	SCO 41
*Casearia combaymensis* Tul.	HUMFOOD: Food (Edible, Fruit)	Ihui Pama / Yobiberoxoco / Chana Jisicato (Ch)	CH106
*Lunania parviflora* Spruce ex Benth.	MEDVET: Digestive system (Diarrhea, Bark; Stomach ache, Bark); Endocrine system (Liver pain, Bark); General Ailments with Unspecific Symptoms (Vomit, Bark and fruit); Musculo-skeletal system (Rheumatism, Bark)	Nishi Tsanóna (Ch); Bejuco / Chacaka (Sp)	DOA 13
Sapindaceae			
*Matayba scrobiculata* (H.B.K.) Radkl.	FUEL: Firewood (Firewood - Caro, Trunk)	Sama negra (Sp)	CH107
*Paullinia* sp.	FUEL: Firewood (Firewood - Caro, Trunk); HUMFOOD: Food (Edible, Fruit); UTEN&TOOL: Hunting & fishing tools (Barbasco - Axa, Trunk)	Shoshapo (Ch); Barbasco / Muela (Sp)	DOA 27
*Serjania lethalis* A. St. Hill	CONST: Houses (Frame house, Trunk; Tirante - Cano bëpotó, Trunk); FUEL: Firewood (Firewood - Caro, Trunk); HUMFOOD: Food (Edible, Fruit); MEDVET: Digestive system (Diarrhea, Bark; Stomach ache, Bark); General Ailments with Unspecific Symptoms (Vomit, Bark); UTEN&TOOL: Hunting & fishing tools (Barbasco - Axacoro, Trunk); Labour tools (Sandpaper, Leaf)	Axa Coro / Carahina Nihi (Ch); Barbasco (Sp)	CH108
*Serjania pyramidata* Radkl.	CULT: Personal adornment (Ornament - Maxëití, Fruit)	Capë Itsa (Ch)	CH109
*Serjania* sp.	UTEN&TOOL: Hunting & fishing tools (Barbasco - Axacoro, Trunk)	Axa Coro (Ch); Barbasco (Sp)	DOA 6, SCO 33
*Talisia acutifolia* Radkl.	HUMFOOD: Food (Edible, Fruit); UTEN&TOOL: Labour tools (Shovel, Trunk)	Pitón (Sp)	CH110
Sapotaceae			
*Chrysophyllum sparsiflorum* Klotzsch ex Miq.	FUEL: Firewood (Firewood - Caro, Trunk)	Quishpi (Ch); Quispi (Sp)	CH111
*Micropholis guyanensis* (A.DC.) Pierre	FUEL: Firewood (Firewood - Caro, Trunk); HUMFOOD: Food (Edible, Fruit)	Toro Quirihua (Ch)	MOV 61
*Micropholis guyanensis* cf. (A.DC.) Pierre	HUMFOOD: Food (Edible, Fruit)	Coquino (Ch)	CH112
*Micropholis lanceolata* (C. Martius & Eichler) Pierre	HUMFOOD: Food (Edible, Fruit)	Bimi Muishi (Ch)	CH113
*Pouteria caimito* (Ruiz & Pav.) Radlk.	FUEL: Firewood (Firewood - Caro, Trunk); HUMFOOD: Food (Edible, Fruit)	Quëo (Ch)	CH114
*Pouteria lucuma* (Ruiz & Pav.) Radlk.	HUMFOOD: Food (Edible, Fruit)	Quëo (Ch); Lucuma (Sp)	CH115
*Pouteria macrophylla* (Lam.) Eyma	HUMFOOD: Food (Edible, Fruit); MEDVET: General Ailments with Unspecific Symptoms (Vomit, Bark); Skin and subcutaneous tissue (Puchichi)	Yahë (Ch)	CH116
*Pouteria nemorosa* Baehni	HUMFOOD: Food (Edible, Fruit); MEDVET: Infections and infestations (Scabies); Insect and athropod bites (Insectbite); UTEN&TOOL: Domestic utensils (Basket - Nishicacano, Bark)	Bata Jihui / Batabí (Ch); Coquino (Sp)	CH117
*Pouteria ramiflora* (Mart.) Radkl.	CONST: Houses (House post- Jibamë, Trunk; Xano, Trunk); FUEL: Firewood (Firewood - Caro, Trunk); UTEN&TOOL: Domestic utensils (Batán - Xaxo, Trunk); Transportation (Canoe, Trunk)	Xanë Yobini (Ch); Tajibo blanco / Almendrillo blanco (Sp)	MOV 12
Simaroubaceae			
*Simarouba amara* Aubl.	CONST: Houses (To tie house, Bark); MEDVET: Digestive system (Diarrhea, Bark; Stomach ache, Bark and fruit); Endocrine system (Gallbladder, Bark; Liver pain, Bark and leaf); General Ailments with Unspecific Symptoms (Vomit, Bark); Infections and infestations (Malaria and fever, Bark); Skin and subcutaneous tissue (Caracha, Bark); Urinary system (Kidneys, Bark); UTEN&TOOL: Domestic utensils (Basket - Bano, Bark)	Tarari (Ch); Palo Amargo (Sp)	GOS 6, GCM 2
Siparunaceae			
*Siparuna guianensis* Aubl.	CULT: Ritual (Fragile children, Bark); MEDVET: Digestive system (Diarrhea, Bark); General Ailments with Unspecific Symptoms (Vomit, Bark); Infections and infestations (Malaria and fever, Leaf); Insect and athropod bites (Buna bite, Bark; Insectbite, Bark); Respiratory system (Cold and flu, Leaf); Skin and subcutaneous tissue (Caracha); Snakebites and Ray stings (Sankebites); UTEN&TOOL: Domestic utensils (Smoke to mosquito repelent)	Shisho Itsa / Xaba ghishu itsa / Xabá shishohitsa (Ch)	BCM 13, GCM 4, GOS 5, MOA 8, MSM 3, 11, SCO 8
*Siparuna krukovii* A.C. Sm.	FUEL: Firewood (Firewood - Caro, Trunk); MEDVET: Infections and infestations (Smallpox, Bark); Insect and athropod bites (Buna bite, Bark; Insectbite, Bark)	Shisho Itsa / Xëto itsa (Ch)	DOA 19, MOV 32, RBU 17829
*Siparuna* sp.	MEDVET: Urinary system (Kidneys, Root)	Xabá shishohitsa (Ch)	BCM 3
Smilacaceae			
*Smilax flavicaulis* Rusby	MEDVET: Digestive system (Diarrhea)	Cayú (Sp)	RBU 17861
*Smilax poeppigii* Kunth.	MEDVET: Digestive system (Diarrhea, Young leaf)	Patiari jomoxa (Ch); Guayaba (Sp)	CH118
*Smilax* sp.	MEDVET: Urinary system (Kidneys, Leaf, root and whole plant)	Yahuaxë (Ch)	JSM 18
Solanaceae			
*Capsicum annuum* L.	HUMFOOD: Food (Edible, Fruit); MEDVET: Skin and subcutaneous tissue (Puchichi, Leaf)	Aji / Aji dulce / Aji rojo (Sp)	
*Cestrum strigillatum* Ruiz & Pav.	MEDVET: Skin and subcutaneous tissue (Caracha, Bark)	Yahua taho (Ch)	CH119
*Lycianthes glandulosa* (Ruiz. & Pav.) Bitter	CULT: Personal adornment (Ornament - Maxëití, Fruit; Ornament - Shinoxëta, Fruit); HUMFOOD: Food (Edible, Fruit)	Bimi Chëxë (Ch)	CH120
*Lycopersicon esculentum* Mill.	HUMFOOD: Food (Edible, Fruit)	Tomate (Sp)	
*Nicotiana rustica* L.	MEDVET: Cultural diseases and disorders (Bad air and scare - Ratëaina, Leaf); Infections and infestations (Boro, Leaf); Reproductive system and sex health (Menstrual pain, Leaf); Sensory system (Earache, Leaf); Skin and subcutaneous tissue (Caracha, Leaf); Snakebites and Ray stings (Sankebites, Leaf)	Romë / Rumë (Ch); Tabaco (Sp)	
*Solanum betaceum* Cav.	MEDVET: Infections and infestations (Malaria and fever, Bark)		RBU 17864
*Solanum lorentzii* Bitter	MEDVET: Dental health (Toothache, Root); General Ailments with Unspecific Symptoms (Headache, Leaf); Vomit, Whole plant (Infections and infestations); Anthelmintic, Bark (Respiratory system); Cold and flu, Bark (Cold and flu, Leaf); Sensory system (Earache, Leaf); Urinary system (Kidney infection, Leaf)	Jimi nihi / Nohini jihui / Nohini nihi (Ch); Uvita (Sp)	BCM 12, GOS 24, MOV 19
*Solanum mammosum* L.	MEDVET: Infections and infestations (Smallpox, Leaf); Skin and subcutaneous tissue (Caracha, Bark)	Popotoa (Ch); ManSilla (Sp)	
*Solanum pensile* Sendtn.	CULT: Ritual (Good luck in fishing)	Cashixopá (Ch)	CH120
*Solanum placitum* C.V. Morton	MEDVET: Cultural diseases and disorders (Bad air and scare - Ratëaina)	Yobini (Ch); Hoja hedionda (Sp)	CH121
*Solanum proteanthum* Bohs	CONST: Houses (Tirante - Cano bëpotó, Trunk); FUEL: Firewood (Firewood - Caro, Trunk); MEDVET: Digestive system (Diarrhea, Trunk); General Ailments with Unspecific Symptoms (Vomit, Trunk)	Shia (Ch)	CH122
*Solanum tuberosum* L.	HUMFOOD: Food (Edible, Root); MEDVET: General Ailments with Unspecific Symptoms (Vomit, Root and whole plant); Infections and infestations (Amoebas, Seeds)	Papa (Sp)	
Staphyleaceae			
*Turpinia occidentalis* subsp. *breviflora* Croat	HUMFOOD: Food (Edible, Fruit); MEDVET: General Ailments with Unspecific Symptoms (Body pain, Root; Headache, Leaf; Pain, Root); Skin and subcutaneous tissue (Haemorrhage)	Jihui Xoco / Strelitziaceae (Ch); Huallabilla de pampa / Papaya (Sp)	CH123
Strelitziaceae			
*Phenakospermum guianensis* Aubl.	CONST: Houses (Hedge - Panë, Trunk; Tie - Xahui, Leaf; Xapocoti, Leaf); Thatch (Huaracha roof, Leaf; Ridgepole - Xobomapatí, Leaf; Roof - Xëhuahacacató, Leaf); CULT: Clothes & accessories (Skirt woman, Leaf); Personal adornment (Ornament - Matsamití, Leaf; Ornament - Maxëití, Leaf; Ornament - Mënëxëtí, Leaf); FUEL: Firewood (Firewood - Caro, Bark); Other fuel (Ceramics - Chomo, Bark); MEDVET: Digestive system (Diarrhea, Exudate; Stomach ache, Exudate); Endocrine system (Liver pain, Exudate); General Ailments with Unspecific Symptoms (Vomit, Exudate); Infections and infestations (Infections, Exudate; Leishmaniasis, Exudate); Respiratory system (Cold and flu, Exudate; Cough, Exudate); Skin and subcutaneous tissue (Burns, Exudate; Caracha, Exudate; Skin fungus, Leaf; Wounds and cuts, Exudate); Snakebites and Ray stings (Sankebites, Exudate); Urinary system (Kidney pain, Exudate; Kidneys, Exudate); UTEN&TOOL: Domestic utensils (Basket - Chichama, Bark; Basket - Nishicacano, Bark; Basket - Purupachi, Bark; Basket - Yamachi, Bark; Fan - Huëquëti, Leaf); Rope (Rope - Rispichi, Leaf); Wrappers; Wrappers, Leaf)	Mani Coro / Manihua (Ch); Patujú (Sp)	
Styracaceae			
S*tyrax* sp.	HUMFOOD: Food (Edible, Fruit)	Ahua Tishi (Ch); Ahuai (Sp)	CH124
Talinaceae			
*Talinum paniculatum* (Jacq.) Gaertn.	MEDVET: Endocrine system (Liver pain, Leaf); General Ailments with Unspecific Symptoms (Headache, Trunk); Musculo-skeletal system (Swelling, Trunk); Respiratory system (Cold and flu, Leaf); Sensory system (Earache, Leaf)	Nohini (Ch)	GOS 18, MOV 23
Tectariaceae			
*Triplophyllum protensum* (Afzel. ex. Sw.) Holttum	MEDVET: Cultural diseases and disorders (Bad air and scare - Ratëaina, Leaf)	Toria huitaxo (Ch); Piñón morado (Sp)	CH125
Thelypteridaceae			
*Thelypteris abrupta* (Desv.) Proctor	MEDVET: Sensory system (Earache, Leaf)	Xëqui jahëhua (Ch)	CH126
Trigoniaceae			
*Trigonia killipii* J.F. Macbr.	MEDVET: Digestive system (Diarrhea, Bark); General Ailments with Unspecific Symptoms (Vomit, Bark); Infections and infestations (Malaria and fever, Bark)	Cashixopá (Ch)	CH127
Ulmaceae			
*Ampelocera edentula* Kuhlm.	CONST: Houses (Muchacho - Ninotí, Trunk; Pasa ratón - Xoya jabatí, Trunk; Ridgepole - Maracatí, Trunk; Roof beam - Canoxoco, Trunk; Tie - Xahui, Bark; Tirante - Cano bëpotó, Trunk; Tirante corto - Cano Bësëcamë, Trunk; Tirante largo - Cano pixquëna, Trunk)	Palo Yodo (Sp)	CH128
*Celtis iguanea* (Jacq.) Sarg.	CONST: Thatch (Roof - Xëhuahacacató, Trunk)	Chichipa (Sp)	CH129
Urticaceae			
*Cecropia ficifolia* Warb. ex Snethl.	CONST: Houses (Hedge - Panë, Trunk; Tie - Xahui, Bark); Thatch (To tie roof, Bark); CULT: Personal adornment (Ornament - Amënoxëta, Leaf; Ornament - Tsirispi, Bark); Recreational (Zampoña - Bistó, Bark); FUEL: Firewood (Firewood - Caro, Trunk); HUMFOOD: Food (Edible, Fruit); MEDVET: General Ailments with Unspecific Symptoms (Vomit, Bark); Infections and infestations (Scabies); UTEN&TOOL: Domestic utensils (Hammock - Nishi, Bark; Pestle of Tacu, Trunk); Hunting & fishing tools (Arrow - Bicobi, Bark; Arrow - Paca, Bark; Arrow - Quërëquë, Bark; Arrow - Tahua Quëspini, Bark; Arrow - Tiopi, Bark; Bow - Canatí, Bark); Labour tools (Planting stick - Xësati, Trunk); Rope (Rope - Rispichi, Bark)	Bocono / Tiopi (Ch); Ambaibo (Sp)	CH130
*Cecropia sciadophylla* Mart.	MEDVET: Respiratory system (Cold and flu)	Bocobí (Ch); Hierba de loro (Sp)	CH131
*Cecropia strigosa* Trécul	FUEL: Firewood (Firewood - Caro, Trunk)	Bocobí (Ch)	JSM 14
*Pourouma cecropiifolia* Mart.	UTEN&TOOL: Labour tools (Planting stick - Xësati, Trunk)	Quëxqui xaquini (Ch)	CH132
*Pourouma guianensis* Aubl.	CONST: Houses (Frame house, Trunk; Jihuixaca, Trunk; Ridgepole - Maracatí, Trunk; Tirante - Cano bëpotó, Trunk; To tie fence, Bark; To tie house, Bark); Thatch (To tie roof, Bark); HUMFOOD: Food (Edible, Fruit)	Xaquini (Ch); Piraquina (Sp)	DOA 21, SCO 23
*Pourouma minor* Benoist	ANIMFOOD: Fodder (Edible, Fruit); FUEL: Firewood (Firewood - Caro, Trunk); UTEN&TOOL: Hunting & fishing tools (Bow - Canatí, Trunk); Labour tools (Sandpaper, Leaf)	Xaquini / Yahë (Ch)	DOA 31, MOV 34
*Urera baccifera* (L.) Gaudich ex Wedd.	MEDVET: Blood and Cardio-vascular system (Heartache, Bark, leaf and root); Infections and infestations (Malaria and fever); Musculo-skeletal system (Rheumatism, Leaf); Respiratory system (Cold and flu, Bark and leaf); Sensory system (Inflammation of eyes, Bark and leaf)	Nahua Shishahua / Pia nihi (Ch); Pega pega / Pica pica (Sp)	DOA 49, GOS 46, JSM 55, MOV 56
Verbenaceae			
*Aloysia triphylla* Royle	MEDVET: Digestive system (Stomach ache, Leaf)	Toronjil (Sp)	
*Lantana cujabensis* Schauer	CONST: Houses (Frame house, Trunk); HUMFOOD: Food (Edible, Fruit); MEDVET: General Ailments with Unspecific Symptoms (Headache, Leaf); Infections and infestations (Malaria and fever, Bark and leaf); Respiratory system (Cold and flu, Flower and leaf)	Bahua Rëxa (Ch); Hierba de loro (Sp)	JSM 19
*Lantana trifolia* L.	MEDVET: Infections and infestations (Malaria and fever, Bark)	Urn (Ch)	CH133
*Lantana* sp.	CONST: Houses (Frame house, Trunk); HUMFOOD: Food (Edible, Fruit); MEDVET: Digestive system (Stomach ache, Fruit and trunk); Endocrine system (Liver pain, Seeds); Skin and subcutaneous tissue (Haemorrhage, Root)	Capëtërëbó (Ch); Biribá / Condura (Sp)	ESR 27
*Petrea* sp. 1	MEDVET: Digestive system (Diarrhea, Bark and leaf; Stomach ache, Bark); General Ailments with Unspecific Symptoms (Vomit, Bark)	Ponochí (Ch); Bejuco (Sp)	GOS 20, JSM 42
*Petrea* sp. 2	MEDVET: Digestive system (Diarrhea, Trunk); General Ailments with Unspecific Symptoms (Vomit, Trunk)	Ponochí (Ch)	SCO 22
*Stachytarpheta cayennensis* (Rich.) Vahl	CULT: Ritual (Crying children); FUEL: Firewood (Firewood - Caro, Trunk); MEDVET: Infections and infestations (Malaria and fever, Leaf)	Camanó Nihi (Ch); Cola de rata (Sp)	ESR 24, GOS 2
*Vitex cymosa* Bert. ex Spreng.	HUMFOOD: Food (Edible, Fruit)	Tarumá (Sp)	CH134
Violaceae			
*Leonia cymosa* Mart.	FUEL: Firewood (Firewood - Caro, Trunk); HUMFOOD: Food (Edible, Fruit)	Mai Rao (Ch)	CH135
*Rinorea guianensis* (Melch.) Ducke	FUEL: Firewood (Firewood - Caro, Trunk); MEDVET: Infections and infestations (Hepatitis, Trunk)	Mai Rao (Ch)	CH136
*Rinoreocarpus ulei* (Melch.) Ducke	CONST: Houses (Muchacho - Ninotí, Trunk); Other constructions (Floor - Machimbre, Trunk); CULT: Recreational (Toys, Seeds); FUEL: Firewood (Firewood - Caro, Trunk); MEDVET: Infections and infestations (Malaria and fever, Bark); Reproductive system and sex health (Abortive, Bark); Skin and subcutaneous tissue (Acne); UTEN&TOOL: Domestic utensils (Furniture, Trunk; Tacú - Arusa timatí, Trunk)	Jihui Joxo / Shihuë / Tapa ristí / Xoquë xëquërë (Ch); Blanquillo / Cafesillo / Toco (Sp)	MOV 39, SCO 24
Indet. sp. 1	FUEL: Firewood (Firewood - Caro, Trunk)	Bëpasti (Ch)	CH136
Vitaceae			
*Cissus erosa* Rich.	CONST: Houses (Frame house, Trunk; Hedge - Panë, Trunk; Jihuixaca, Trunk; Roof beam - Canoxoco, Trunk; Tirante - Cano bëpotó, Trunk; To tie house, Bark); FUEL: Firewood (Firewood - Caro, Trunk); HUMFOOD: Food (Edible, Fruit); MEDVET: Digestive system (Diarrhea, Seeds)	Nai Nishi (Ch); Sirari (Sp)	CH137
*Cissus sicyoides* L.	HUMFOOD: Food (Edible, Fruit); MEDVET: Snakebites and Ray stings (Sankebites)	Carabó Coatí (Ch)	CH138
Vochysiaceae			
*Qualea acuminata* Spruce ex Warm.	CULT: Personal adornment (Ornament - Maxëití, Fruit)	Omaca Bëro (Ch)	CH139
*Qualea grandiflora* Mart.	CONST: Houses (Frame house, Trunk; Jihuixaca, Trunk; To tie house, Bark); HUMFOOD: Food (Edible, Seeds); MEDVET: Skin and subcutaneous tissue (Acne, Seeds)	Almendro (Sp)	RBU 17849
*Qualea paraensis* Ducke	CONST: Houses (Hedge - Panë, Trunk; Muchacho - Ninotí, Trunk; Pasa ratón - Xoya jabatí, Trunk; Ridgepole - Maracatí, Trunk; Roof beam - Canoxoco, Trunk; Tie - Xahui, Bark; Tirante - Cano bëpotó, Trunk; Tirante largo - Cano pixquëna, Trunk); FUEL: Firewood (Firewood - Caro, Trunk); HUMFOOD: Food (Edible, Fruit)	Jihui Sama / Jihui Xoco (Ch); Chocolate / Chocolatillo (Sp)	CH140
*Qualea* sp.	MEDVET: Skin and subcutaneous tissue (Caracha, Bark; Caracha, Root; Puchichi, Bark; Skin infection, Bark)	Mëtëquë (Ch)	CH141
*Vochysia vismiifolia* Spruce ex Warm.	CONST: Houses (Muchacho - Ninotí, Trunk; Pasa ratón - Xoya jabatí, Trunk; Roof beam - Canoxoco, Trunk; Tirante - Cano bëpotó, Trunk; Tirante largo - Cano pixquëna, Trunk); CULT: Other cultural (Crafts, Trunk); HUMFOOD: Food (Edible, Fruit); MEDVET: Digestive system (Stomach ache, Bark)	Canú / Jihui Coshi / Cano (Ch); Cedro (Sp)	CH142
Zingiberaceae			
*Renealmia breviscapa* Poepp. & Endl.	FUEL: Firewood (Firewood - Caro, Trunk); HUMFOOD: Food (Edible, Flower); MEDVET: Insect and athropod bites (Insectbite, Exudate)	Manihua shiri (Ch)	RBU 17854
*Zingiber officinale* Roscoe	HUMFOOD: Food (Edible, Fruit); MEDVET: Cultural diseases and disorders (Bad air and scare - Ratëaina, Root); Dental health (Toothache, Root and trunk); General Ailments with Unspecific Symptoms (Headache, Trunk; Vomit, Whole plant); Skin and subcutaneous tissue (Haemorrhage, Young leaf); Snakebites and Ray stings (Sankebites, Root)	Shibiri (Ch); Gengibre (Sp)	

**Fig. 2:**
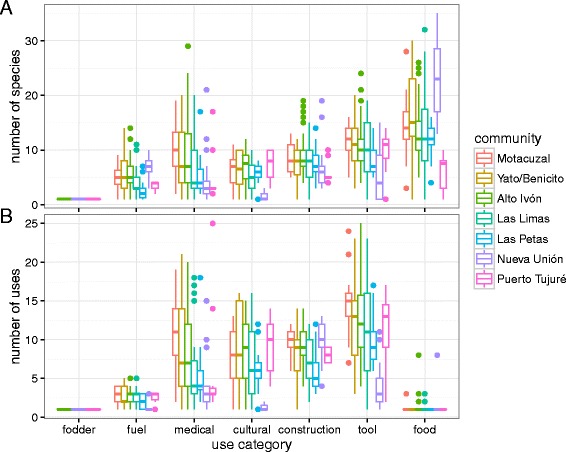
Number of species (**a**) and uses (**b**) reported per interview reported in each use category for each community (N: Motacuzal = 25, Yato Benicito = 51, Alto Ivón = 83, Las Limas = 45, Las Petas = 15, Nueva Unión = 20, Puerto Tujuré = 11. 40 interviews without a community indicated are not shown)

**Fig. 3:**
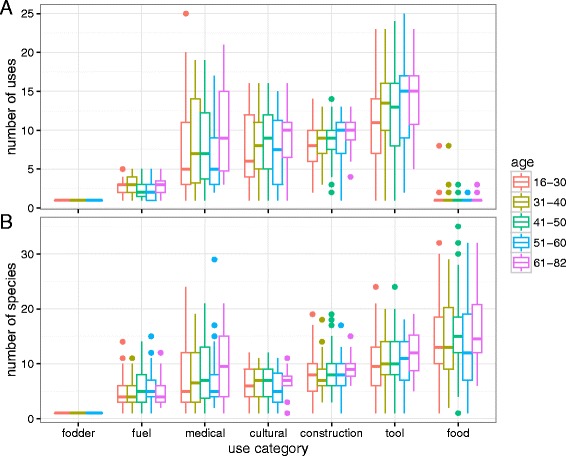
Number of uses (**a**) and species (**b**) reported per interview reported in each use category for each age group (N: 16–30 = 110, 31–40 = 65, 41–50 = 68, 51–60 = 25, 61–81 = 24)

**Fig. 4:**
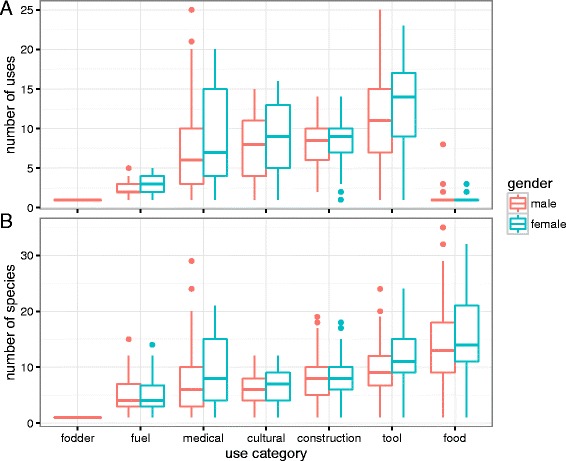
Number of uses (**a**) and species (**b**) reported per interview in each use category for men (*N* = 154) and women (*N* = 138)

The larger Chácobo communities showed very similar patterns in the number of species used, with differences within communities usually greater than between, although Nueva Unión stood out in reporting more food species (Fig 2a). Likewise, all communities were similar in plant–uses (use descriptions for a species within each use category), although in this case Nueva Unión reported fewer use descriptions within the Utensils and tools and Cultural categories, while Motacuzal and Alto Ivón reported more medical uses (Fig. 2b). Within these categories, number of species and uses was fairly consistent across age groups, though we observed a trend for some categories of more species and uses known with increasing age. The age group between 51 and 60 years (i.e. the first age group growing up under missionary rule), showed a slightly lower knowledge, especially evident in the medical and cultural categories but also in food plants (Fig. 3). These metrics are also quite similar across gender, although across most categories the average number of species and uses reported by women was slightly higher (Fig. 4).

### Who uses what and how?

**Fig. 5:**
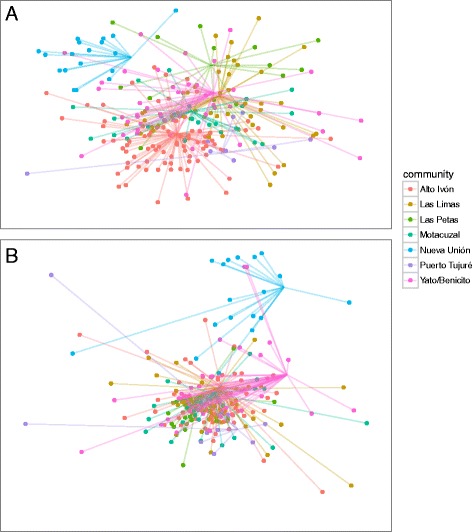
Reports by community for plant species (**a**) and uses (**b**)

Despite the similarities among communities in total species and uses reported, we found that informant community significantly influenced both which plants and which uses individual informants reported (Table 2 a&b). In contrast, and in accord with the results above, age and gender did not significantly influence either. Ethnicity of the participants influenced which plant species they used, but did not explain what they were used for. Given the very low r^2^ values, it is clear that much variety in uses was not explained by any demographic and environmental variables explored (Table 2 a&b). In the ordination, we can see this effect more clearly: although there was much overlap, the communities clearly structure which plants were reported. This difference was however much driven by the reports Nueva Unión (Fig. 5).

**Table 2:** Who uses what, and how?

	r-squared	*p*-value
A. In plant-space (plant species mentioned) ordination		
age	−1.00	0.256
gender	0.00	0.878
ethnicity	0.04	0.015*
community	0.30	0.001***
B. In use-space (uses mentioned for specific species) ordination		
age	−0.96	0.638
gender	0.01	0.174
ethnicity	0.04	0.092
community	0.27	0.001***

* = significant

*** = highly significant

While age did not in itself explain the ordination well, we did find certain plants to be associated with age categories. In this we found no indicator plants or uses among the first three age groups (16–30, 31–40, 41–50), which suggests to some extent that plants and uses reported by these groups are less distinct than that of the second two age groups (51–60, >60). The 51–60 age group was associated with by *Styrax* sp*., Iryanthera juruensis, Xylopia ligustrifolia, Hirtella pilosissima, Inga* sp*.* 1*, and Piper nigrispicum*, while the >60 group was indicated by *Gustavia hexapetala, Astrocaryum aculeatum, Phenakospermum guianensis, Attalea phalerata, Apuleia leiocarpa, Bixa orellana, Hancornia speciosa, Zingiber officinale,* and *Eriotheca* sp. Likewise, the use subcategory Firewood was associated with the 51–60 age group while the medicinal use subcategories: Skin and subcutaneous tissue, Sensory system, Respiratory system and Musculo–skeletal system all were associated with the >60 age group.

Likewise, although gender did not fit to the overall ordinations, there was a large number of plants associated with female, and a much smaller one with male respondents (Table 3).

**Table 3:** Indicator values of species by Gender

species	gender	indicator value	probability
*Hirtella pilosissima*	m	0.11	0.01
*Schefflera morototoni*	m	0.09	0.02
*Xylopia* sp*.*	m	0.07	0.01
*Eschweilera albiflora*	m	0.04	0.05
*Attalea phalerata*	f	0.50	0.01
*Gossypium barbadense*	f	0.49	0.01
*Phenakospermum guianensis*	f	0.47	0.03
*Licania octandra* subsp*. pallida*	f	0.45	0.01
*Apeiba tibourbou*	f	0.44	0.01
*Capirona decorticans*	f	0.43	0.00
*Cecropia ficifolia*	f	0.41	0.00
*Musa x paradisiaca*	f	0.39	0.04
*Helicostylis tomentosa*	f	0.38	0.01
*Omphalea diandra*	f	0.35	0.01
*Uncaria guianensis*	f	0.35	0.04
*Bixa orellana*	f	0.35	0.00
*Manihot esculenta*	f	0.31	0.03
*Ficus* sp*.*	f	0.30	0.04
*Tabebuia sp.*	f	0.30	0.01
*Oryza sativa*	f	0.29	0.04
*Brosimum utile* subsp*. ovatifolium*	f	0.29	0.03
*Amburana cearensis*	f	0.28	0.03
*Pouteria ramiflora*	f	0.28	0.00
*Dioscorea latifolia*	f	0.23	0.02
*Piper piscatorum*	f	0.22	0.00
*Xanthosoma sagittifolium*	f	0.18	0.00
*Nicotiana rustica*	f	0.18	0.00
*Woytkowskia spermatochorda*	f	0.18	0.00
*Styrax sp.*	f	0.18	0.00
*Xanthosoma striolatum*	f	0.17	0.00
*Persea americana*	f	0.17	0.02
*Bryophyllum* sp*.*	f	0.15	0.00
*Chrysophyllum sparsiflorum*	f	0.14	0.00
*Erythroxylum coca*	f	0.13	0.00
*Micropholis guyanensis*	f	0.09	0.04
*Myrcia regnelliana*	f	0.08	0.00
*Qualea paraensis*	f	0.06	0.03

Interestingly, all indicator uses were exclusively associated with women (Table 4).

**Table 4:** Indicator uses and gender association

Use subcategory	gender	indicator value	probability
Domestic utensils	f	0.53	0.01
Personal adornment	f	0.51	0.00
Other fuel	f	0.44	0.02
Clothes & accessories	f	0.43	0.01
Skin and subcutaneous tissue	f	0.42	0.00
Snakebites and Ray stings	f	0.28	0.01
Dental health	f	0.27	0.02
Cultural diseases and disorders	f	0.25	0.00
Insect and arthropod bites	f	0.24	0.02
Endocrine system	f	0.24	0.04

### Informant consensus factors (ICF)

Looking at specific use categories we found broadly similar trends across age categories and genders: tool, construction and food uses usually had the most use reports. We found a lower number of medicinal use reports, although the same number of respondents reported medicinal uses. Food uses consistently had less ICF than tool and construction uses, and medicinal uses even less. Cultural uses, while often reported by fewer informants and with fewer uses, show disproportionately high ICF (Table 5).

**Table 5:** Who uses what, and how: informant consensus factor

age	gender	Nur use reports	informants	species	mean ICF across use categories	ICF sd acrossuse categories
16–30	male	3517	55	213	0.90	0.06
16–30	female	3571	44	188	0.92	0.04
31–40	male	1834	29	181	0.83	0.07
31–40	female	2529	31	176	0.89	0.06
41–50	male	2314	32	192	0.86	0.06
41–50	female	2317	31	180	0.88	0.06
51–60	male	1146	15	167	0.77	0.10
51–60	female	562	8	123	0.68	0.12
61–82	male	747	10	142	0.72	0.13
61–82	female	1150	14	147	0.80	0.09

Plant relative importance metrics did show a different picture underlining the problems of using diversity indices. The Cultural Importance Index yielded wildly different species sets for Community and Individuals, and both Use Value Index and Use–diversity Index again yielded different sets as species as most important (Table 6).

**Table 6:** Plant importance metrics

Top species by CIcom	CIcom	UV	CIinf	Du
* Euterpe precatoria*	**4.78**	1.37	1.89	**1.97**
* Gossypium barbadense*	**3.86**	**2.54**	1.98	1.26
* Attalea phalerata*	**3.82**	**2.64**	**2.14**	1.48
* Xylopia peruviana*	**3.77**	**2.97**	**2.12**	1.23
* Phenakospermum guianensis*	**3.76**	**1.77**	1.76	1.60
Top species by UV	UV	CIcom	CIinf	Du
* Vismia macrophylla*	**4.20**	2.73	1.55	0.59
* Xylopia peruviana*	**2.97**	**3.77**	**2.12**	1.23
* Attalea phalerata*	**2.64**	**3.82**	**2.14**	1.48
* Gossypium barbadense*	**2.54**	**3.86**	1.98	1.26
* Bactris gasipaes*	**2.50**	1.91	1.24	0.39
* Astrocaryum aculeatum*	**2.28**	2.96	1.44	1.02
* Attalea maripa*	**1.96**	3.33	1.87	1.46
* Gynerium sagittatum*	**1.95**	2.41	1.57	0.87
* Phenakospermum guianensis*	**1.77**	**3.76**	1.76	1.60
* Licania octandra* subsp*. pallida*	**1.73**	1.81	1.25	0.45
Top species by Du	Du	CIcom	CIinf	UV
* Cedrela fissilis*	**2.10**	3.17	1.24	0.16
* Citrus aurantiifolia*	**2.05**	2.40	1.06	0.13
* Croton* sp*. 1*	**2.02**	1.57	1.10	0.04
* Jatropha gossypiifolia*	**2.00**	2.17	1.64	0.08
* Euterpe precatoria*	**1.97**	**4.78**	1.89	1.37
* Hymenaea courbaril*	**1.82**	3.00	1.17	0.32
Top species by CIinf	CIinf	CIcom	UV	Du
* Piper peltatum*	**3.00**	3.00	0.01	1.10
* Attalea phalerata*	**2.14**	**3.82**	**2.64**	1.48
* Xylopia peruviana*	**2.12**	**3.77**	**2.97**	1.23

Most important species in each index in bold

**Fig. 6:**
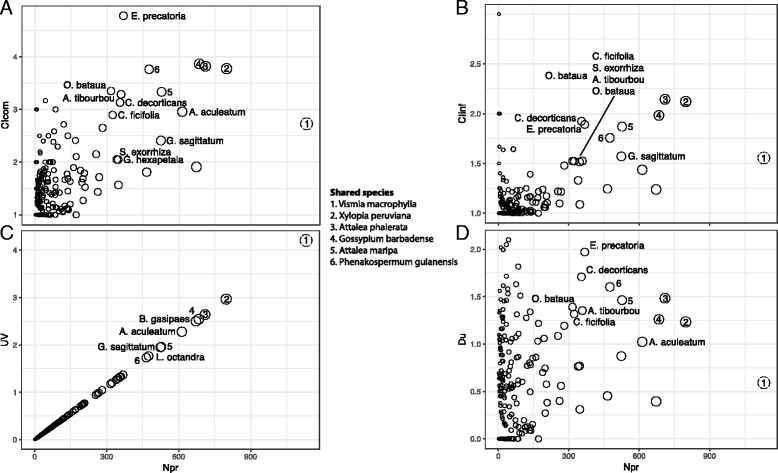
Species sized by number of reports (Npr) plotted against four metrics of importance: Community Cultural Importance (**a**), Informant Cultural Importance (**b**), Use Value (**c**), and Diversity of Uses (**d**). The most important species (top right quadrant) are labelled with their names, and the six shared across all four metrics are numbered

**Fig. 7:**
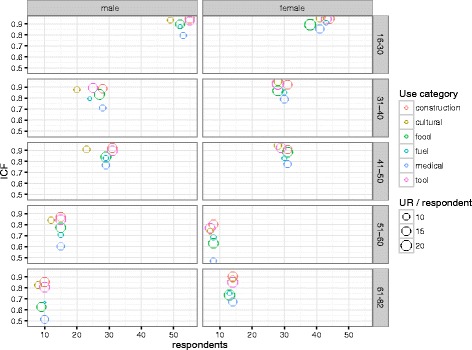
Informant consensus factor (ICF) for each use category, among male and female respondants of each age group. Points are sized by the number of use reports (UR) per respondent

Because the Cultural Importance Index tends to prioritize species with few informants, we highlighted the species that had both high index values in general, and also a large number of reports to elucidate species that were of high importance in all indices. As result, *Vismia macrophylla*, *Xylopia peruviana*, *Attalea phalerata*, *Gossypium barbadense*, *Attalea maripa* and *Phenakospermum guianensis* were elucidated as the most important species in the daily life of the Chácobo community (Fig. 6). Overall, however, informant consensus was very high in across all age groups and across all use categories (Fig. 7). Arecaceae, Fabaceae, Malvaceae and Rubiaceae were found to be the most important plant families used across most indices, although Moraceae did yield a higher ranking in Use Value (Table 7).

**Table 7:** What is used? (plant relative importance metrics by family)

Family	CIinf	CIcom	UV	Du
Arecaceae	**5.1**	**8.7**	**14.5**	**1.9**
Fabaceae	**3.4**	**6.9**	**5.0**	**2.0**
Malvaceae	**3.1**	**6.9**	**5.0**	**1.9**
Rubiaceae	**3.0**	**6.4**	**2.9**	**2.3**
Poaceae	**2.5**	**5.5**	**3.9**	1.6
Moraceae	**2.5**	**4.8**	**5.1**	1.3
Annonaceae	**2.3**	**4.1**	**3.5**	1.4
Chrysobalanaceae	1.9	3.4	**2.6**	1.2
Euphorbiaceae	1.9	**4.4**	**2.0**	**1.9**
Strelitziaceae	1.8	3.8	1.8	1.6
Apocynaceae	1.7	3.4	1.2	**1.9**
Talinaceae	1.7	2.0	0.0	1.3
Lecythidaceae	1.6	3.8	1.6	1.3
Urticaceae	1.6	3.2	1.2	1.4
Hypericaceae	1.6	2.7	**4.2**	0.6
Bignoniaceae	1.5	3.4	1.2	**1.8**
Caryocaraceae	1.5	1.5	0.0	1.1
Sapotaceae	1.4	2.2	1.0	1.0
Simaroubaceae	1.3	2.0	0.2	1.3
Rutaceae	1.3	3.2	1.1	1.7
Anacardiaceae	1.3	3.1	0.8	1.3
Meliaceae	1.2	3.2	0.2	**2.1**
Costaceae	1.2	2.3	0.3	1.5
Lamiaceae	1.2	2.6	0.3	1.3
Aristolochiaceae	1.2	2.0	0.2	1.1
Verbenaceae	1.2	2.3	0.4	1.6
Piperaceae	1.2	2.1	0.4	1.0
Burseraceae	1.2	1.4	0.1	0.5
Melastomataceae	1.2	1.7	0.6	0.7
Myrtaceae	1.2	1.7	0.2	1.1
Solanaceae	1.1	2.4	0.3	1.4
Sapindaceae	1.1	1.8	0.5	0.7
Flacourtiaceae	1.1	1.3	0.1	0.9
Cyperaceae	1.1	1.9	0.2	0.9
Crassulaceae	1.1	1.6	0.2	0.5
Zingiberaceae	1.1	1.6	0.1	1.3
Malpighiaceae	1.1	1.3	0.0	1.2
Amaryllidaceae	1.1	1.8	0.0	**1.9**

Most important species in each index in bold

**Fig. 8:**
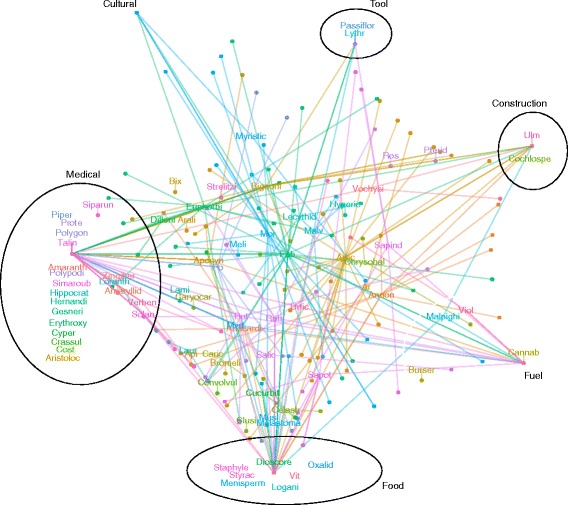
Assemblage of by plant family (r–squared = 0.39, *p* = 0.001)

Results also indicated that qualities of plants did to a certain extent explain which uses they were put to. A large number of plant families had specifically Medicinal uses, while other sets of plant families were specifically used for Food, Utensils and tools, and Construction. Not surprisingly, data also revealed that plant families with high importance in all indices calculated (Arecaceae, Fabaceae, Malvaceae and Rubiaceae) had uses in all categories (Fig. 8).

**Fig. 9:**
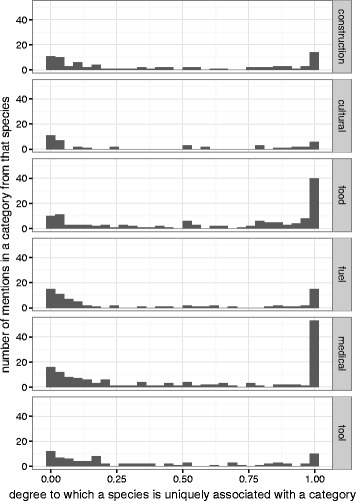
Number of mentions that of a species plotted against that species’ fidelity to the given use category

Different use categories also had different levels of fidelity in the species that were reported for them. For instance, relatively few mentions in the construction and tool categories were of species that are uniquely associated with those categories. In contrast, a much greater proportion of mentions for medical uses were of species that were only used for medical uses. This pattern was also true of food plants (Fig. 9).

### Does language influence use knowledge?

**Fig. 10:**
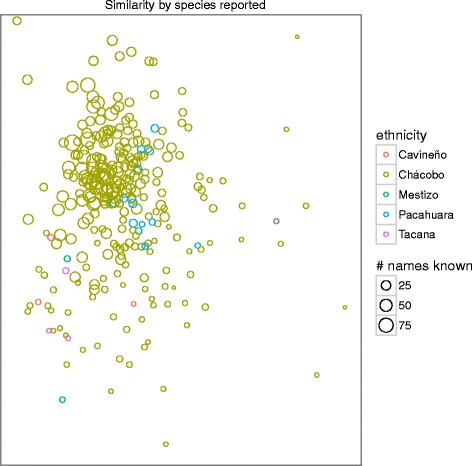
Chácobo language proficiency (point size) plotted onto the ordination of informants in plant space

**Fig. 11:**
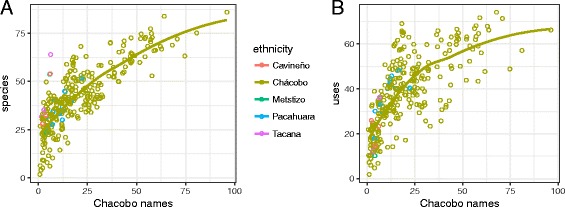
The number of species (**a**) and uses (**b**) was significantly greater for those who reported more Chácobo names. Loess regression line (for Chácobo informants only) is shown

Interviewees who reported more Chácobo names did indeed tend to report more similar sets of knowledge, and knew more species and uses (Fig. 10). In addition, the number of plants or number of uses reported strongly increased with the number of Chácobo names participants knew (Fig. 11). Although in some degree this was a feature of the study (there was no way to informants to report more names than species), it was clear that very few of those participants with great knowledge of species or uses failed to report a large number of Chácobo names.

## Discussion

While other studies found indecisive patterns of the influence of age, or accessibility to markets on traditional knowledge (negative [17, 23, 44, 55]; positive [25, 56, 57]), our study did not reveal any pattern that would link differences in plant–use knowledge to age or accessibility of a location, but simply to specific location and associated flora in each of the communities. In most communities the contact with nature still remains vital to the acquisition of knowledge [58, 59], and the facility to observe and identify the useful plants clearly adds to this.

The observation that local and indigenous languages often package rich traditional ecological knowledge has led to the question in many studies of whether indigenous language abilities influence plant knowledge, i.e. if native language speakers have a higher knowledge than participants only speaking a mainstream language [44, 60]. In our study, the link between language proficiency and other metrics of traditional knowledge (plants and uses reported) does support at least the correlation of these variables, and suggest the possibility of simultaneous language and knowledge retention (or erosion).

The general trend found in relation to the difference in intergenerational knowledge suggests that any patterns are most likely a result of both knowledge transmission, as well as in situ learning, and be related to the time during which people acquire and use knowledge, with the older informants taking more responsibility in their households, who have a need to learn and apply their knowledge [27, 54, 61]. The knowledge of older people might not have been affected by the need to find new subsistence activities, and was thus preserved without external influence [62]. The fact that the only generation that did show decrease of traditional knowledge (albeit slight) was the generation of 41–50-year old participants, who had grown up under restrictive missionary rule, is noteworthy.

The hypothesis that people who are relatively isolated from the market economy share more traditional knowledge than people who live close to cities or larger towns [25], was not met in our study, because in most places the contact to nature still remains vital to the acquisition of knowledge [58, 59]. The predominance of the use for Human food in the more widely shared knowledge can be explained as a long and constant learning process that begins in early childhood, and is common in the more remote locations [26, 54].

There is no doubt that Chácobo daily life has changed in the course of the last century. Early accounts of the Chácobo all indicate the wide use of bark–cloth, and little enthusiasm for the rather conservative clothing style which missionaries tried to introduce [2, 5]. Boom [7] mentions however the complete disappearance of this custom. However, while the Chácobo use western style clothing available in the markets of Riberalta, traditional bark cloth is still widely used for cultural purposes, and most participants knew how to make it.

Changes in the use of traditional implements were very subtle. Most households still use large pounding tubs, as well as the large wooden boards used to pound food, which have not changed over time. Large clay pans for roasting jibe (*Manihot* flour), and smaller ceramic pots are also widely used. Even little stools from the petioles of *Mauritia flexuosa* and balsa wood (*Ochroma* sp.), first documented by Nordenskjöld [1] are still found in many houses, although they were completely missed in all previous studies. The production of burden baskets has not changed since [1], and the same species are still used today. However, only a few older women in the communities still have the skills to weave baskets, and modern implements like backpacks are clearly replacing traditional materials. Similarly, canoes are still an important means of transportation. However, while Nordenskjöld, Haenke and Kelm described canoes made from bark [1, 2, 5], the modern variety is made of hollowed out tree trunks, which is already indicated in [7]. House construction and roofing have however not changed much in the last 100 years. Bows and arrows are still maintained as hunting implements, especially for fishing, and all arrow types found in previous studies are still used among the population, although 22 caliber rifles and 20 gauge shotguns are favored for hunting.

Based on previous reports, we originally hypothesized that many household artifacts as well as traditional clothing had disappeared from Chácobo life. Many of these artifacts were mentioned in the 1922–1970 accounts, but not in later studies. Boom [7] and Bergeron [8] in particular indicate that traditional tools and clothing had disappeared. This turned out to be an interview artifact. Early anthropologists, who focused on Chácobo daily life [2, 5], while Boom and Bergeron focused only on plants collected from one 1 ha forest plot [7, 8]. Our combined study indicates that in fact most artifacts of the Chácobo are still known, and also used, by a large part of the population. This includes traditional clothing that is still being prepared and used on important occasions, as well as hunting and household implements. In daily life however, no traditional clothing and ornaments are found anymore, and the large monkey tooth breast–plates mentioned by [1] and [2] have indeed disappeared.

In case of food, market access has indeed had an influence in Chácobo life. In the 1980’s cassava (*Manihot esculenta*, Euphorbiaceae) was clearly the most important food for Chácobo, and seven varieties were planted (Boom 1987). Maize (*Zea mays*), was planted on 18% of the land, and upland rice (*Oryza sativa*) was only planted on 7% of the land [7]. Nowadays rice has become the staple food of the Chácobo, leaving cassava and maize in a more secondary role, However, all original traditional maize and cassava varieties, as well as traditional banana varieties, are still grown. In our work we also found all edible species mentioned by Boom (1987) as planted in home– and forest–gardens, but the Chácobo had incorporated many additional species, e.g. lemon (*Citrus sinensis*, Rutaceae) in home gardens, and *Psidium*, *Myrica* sp. and *Eugenia* sp. in the forest gardens. One noteworthy exception was the palm Huanimá (*Bactris gasipaes* var*. chichagui*, Arecaceae), actively sown formerly in abandoned clearings to collect palm fruits [7]. In 2015 the palm was only found rarely around the villages, and was no longer planted.

The Chácobo keep using a large number of plants for medicinal purposes although missionaries of the Summer Institute of Linguistics tried to eradicate traditional medicinal plant use and traditional agricultural practices, because they regarded this as pagan [4]. Early anthropological and missionary accounts mentioned hardly any medicinal species [2, 4, 5], but this was clearly an interview artifact. Of the 360 plant species collected by Boom, 174 species were of medicinal value [7]. Bergeron recorded 399 useful plant species, of which 166 were classified as medicinal [8]. This compares favorably to the over 331 useful plant species elucidated in the current study. The Chácobo still favor the preparation of remedies by boiling the leaves, bark or fruits to cure diseases. While Boom did not find a true “healer” among the Chácobo [7], several Chácobo healers were identified in the present study. The knowledge of medicinal plants was particularly alive among older informants interviewed, but younger participants still retained much of such knowledge. The use of plant poisons, especially for fishing was mentioned as highly important by [7], and is still practiced today.

One of the most profound changes in Chácobo life seems to be a return to nomadic patterns, now mostly linked to commerce and income generation. The production of oil from the seeds of Brazil nuts (*Berthollettia excels)* was reported by Boom [7], but is little practiced nowadays – all nuts are now sold to large companies in Riberalta. The Brazil nut harvest takes place from January and March, and during that time now almost the entire Chácobo population migrates to the South of the territory where the largest concentration of *Berthollettia* is encountered. During the rest of the year Alto Ivón remains the main population center. However, many Chácobo have “second” homes in Tokyo, where most of the fields are located at present, or in Triangulo, closer to their main fishing sources, and conveniently located at the road to Riberalta.

## Conclusions

In this paper we illustrate the complexity of perspectives on knowledge at different ages, and the persistence of knowledge over almost a century. We found that traditional knowledge was only partially affected by the processes of exposure to a market economy, and that different knowledge domains experienced different trends as a result of these changes. Overall knowledge was widely distributed, similar to [63]. However, we did not observe a directional knowledge loss, contrasting [64].

We stress the importance to not directly conclude processes of knowledge loss, cultural erosion or acculturation when comparing the knowledge of different age groups. These results should be treated with caution, because they cannot rule out the role of other variables affecting knowledge, including changes in the composition of other important factors that might be affected by the influence of access to a market economy. It is important to remember that learning, and accumulating experiences, require time. For this reason, the alternative explanation that the knowledge of older people tends to have accumulated over time, compared to the younger generation, should also be considered. It also needs to be taken into account that older generations might have different perceptions of their environment, because their points of reference are different from those of younger people. The ability to generate and apply knowledge in human populations enables actions and adjustments in response to current and future changes. Similarly, the ability to generate and apply knowledge, and not the knowledge itself, helps to increase the resilience of socio–ecological systems.

The analysis presented here clearly suggests that perceived knowledge “loss” might easily be an artifact of the researcher’s presence, of limited time, and of a very limited number of participants. Training local interviewers provides an excellent tool yield more reliable information on traditional knowledge and its potential loss in the future.

In compliance with the Nagoya Protocol, the original field notebooks, as well as the complete dataset, and a guide on useful plants of the Chácobo was repatriated to the Chácobo [65]. All members of the tribe have access to the compiled interview data for purposes of learning and education. The data collected are a valuable resource to the community as a tool to preserve their traditional knowledge, and will encourage the launch of research projects and community activities so the information does not become static. Species identified as being most important to the community can be targeted for conservation and restoration activities.
